# Time‐of‐Day Variation in Airway Hyperresponsiveness in Asthma: Potential Circadian Mechanisms and Translational Implications

**DOI:** 10.1002/clt2.70221

**Published:** 2026-11-07

**Authors:** Chang Zhou, Xinyue Hu, Lijuan Zeng, Yi Tang, Chuxian Yi, Guomin Zhang, Sujuan Peng

**Affiliations:** ^1^ The Second Affiliated Hospital of Hunan University of Chinese Medicine Changsha Hunan China

**Keywords:** airway hyperresponsiveness, asthma, chronotherapy, circadian rhythm, temporal regulation

## Abstract

Airway hyperresponsiveness (AHR) is a key physiological feature of asthma and contributes to excessive airway narrowing in response to diverse stimuli. Although asthma‐related airway physiology exhibits marked temporal variation, whether AHR itself is independently regulated by the endogenous circadian clock remains unclear. In this narrative review, we summarize current evidence on the definition and temporal phenotype of AHR, with particular emphasis on distinguishing clock‐time variation from endogenous circadian regulation and on direct evidence from bronchial provocation studies. We further discuss potential mechanisms linking circadian biology to AHR, including neuroendocrine and autonomic gating, epithelial–immune temporal coupling, airway smooth muscle reactivity, and metabolic and redox regulation. Current evidence supports substantial time dependence of asthma‐related airway physiology, whereas direct evidence for an intrinsic circadian rhythm of AHR in humans remains limited with mechanistic insights derived predominantly from animal, ex vivo, and cellular studies. We also review time‐dependent treatment strategies, particularly inhaled corticosteroid (ICS) administration, while distinguishing clinically investigated dosing schedules from experimental clock‐targeted interventions. Overall, circadian biology provides a framework for understanding temporal variation in AHR and may inform future approaches to phenotyping and treatment timing; however, circadian‐based AHR management and clock‐targeted therapies remain investigational and require validation in well‐controlled human studies.

## Graphical abstract

Graphical abstract Biological time, time‐dependent airway hyperresponsiveness, and clinical translation. The external day–night cycle entrains the central clock in the suprachiasmatic nucleus and peripheral pulmonary clocks, whereas sleep and activity, posture, environmental exposures, and medication timing modify the airway state observed at a given clock time. Temporal changes in autonomic and endocrine balance, epithelial–immune activity, airway smooth‐muscle function, and redox regulation may produce different responses to an otherwise standardized bronchial stimulus, expressed as changes in response threshold, dose–response slope, or maximal airway narrowing. Human studies support a clock‐time‐dependent AHR phenotype, but direct evidence for endogenous circadian regulation of AHR remains insufficient. Clinical translation should therefore prioritize standardized recording of test time and medication withholding, followed by phase‐informed and phenotype‐specific management. Human trial evidence is relatively strongest for inhaled corticosteroid timing and selected nocturnal outcomes, whereas direct AHR outcomes remain limited. Evidence for timed bronchodilators, leukotriene modifiers, and biologics is limited or indirect, and clock‐targeted drugs and circadian‐alignment interventions remain exploratory or preclinical. Accordingly, current evidence does not support a single universal treatment time for all patients. Dark blue denotes an observed human phenotype, teal denotes human trial or supportive evidence, orange denotes limited or indirect evidence, and dashed gray denotes exploratory or preclinical approaches. AHR, airway hyperresponsiveness; ASM, airway smooth muscle; ICS, inhaled corticosteroid; PC_20_, provocative concentration causing a 20% decrease in forced expiratory volume in 1 s; PD_20_, provocative delivered dose causing a 20% decrease in forced expiratory volume in 1 s; SCN, suprachiasmatic nucleus.

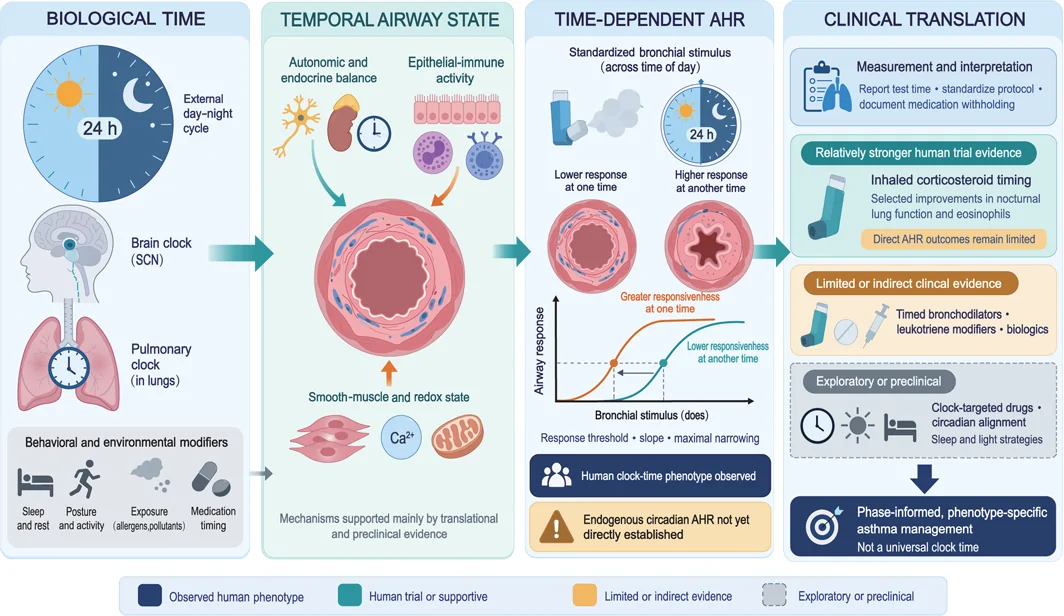

**Abbreviations**
AHR: airway hyperresponsiveness
AMP: adenosine 5′‐monophosphate
ASM: airway smooth muscle
BMAL1: brain and muscle ARNT‐like protein 1
cAMP: cyclic adenosine monophosphate
CLOCK: circadian locomotor output cycles kaput
CRY1: Cryptochrome‐1
EDN: eosinophil‐derived neurotoxin
FeNO: fractional exhaled nitric oxide
FEV1: forced expiratory volume in 1 s
GPX4: glutathione peroxidase 4
GR: glucocorticoid receptor
HDM: house dust mite
HPA: hypothalamic–pituitary–adrenal
ICS: inhaled corticosteroid
IL‐25: interleukin‐25
IL‐33: interleukin‐33
ILC2s: group 2 innate lymphoid cells
IP3Rs: inositol 1,4,5‐trisphosphate receptors
LABAs: long‐acting β2‐agonists
LAMAs: long‐acting muscarinic antagonists
LTRAs: leukotriene receptor antagonists
MAPK: mitogen‐activated protein kinase
MLCP: myosin light chain phosphatase
NHANES: National Health and Nutrition Examination Survey
Nrf2: nuclear factor erythroid 2‐related factor 2
PEFR: peak expiratory flow rate
PER1: Period circadian protein homolog 1
PER2: Period circadian protein homolog 2
ROS: reactive oxygen species
SLC7A11: solute carrier family 7 member 11
SOCE: store‐operated calcium entry
TRPV1: transient receptor potential vanilloid 1
TSLP: thymic stromal lymphopoietin

## 1 Introduction

Asthma is a heterogeneous disease characterized by variable respiratory symptoms and variable expiratory airflow limitation. Airway hyperresponsiveness (AHR) is a key physiological feature of asthma and an important basis for excessive airway narrowing in response to a variety of stimuli [1, 2]. The clinical manifestations of asthma exhibit marked temporal variation, with symptoms and airflow limitation often worsening at night or in the early morning [3]. Repeated bronchial challenge studies have further shown that, in some patients with asthma, the threshold for airway responsiveness varies according to the time of testing, and these changes do not necessarily parallel fluctuations in baseline forced expiratory volume in 1 s (FEV_1_) [4, 5]. These findings suggest that AHR may exhibit time‐dependent variation in a subset of patients with asthma. Consequently, a single conventional daytime assessment may not fully capture the airway responsiveness phenotype, and the timing of testing may also influence the comparison and clinical interpretation of AHR measurements.

Time‐dependent AHR refers to changes in the threshold or magnitude of airway responses to the same stimulus over time. However, differences observed at different clock times cannot, by themselves, establish that these changes are driven by an endogenous circadian rhythm. Under real‐world conditions, temporal variation may be influenced by multiple factors, including sleep, posture, physical activity, food intake, environmental exposures, and the timing of medication administration [6]. Human studies using constant‐routine and forced‐desynchrony protocols have shown that, after controlling for sleep and other behavioral and environmental factors, baseline airway function, symptoms, and the need for rescue medication in patients with asthma still worsen during the biological night, supporting an independent contribution of the endogenous circadian system to asthma‐related airway status [7]. However, bronchial challenge testing was not repeated at different circadian phases in that study; therefore, it could not directly establish that human AHR itself is independently regulated by the endogenous circadian system.

Recent studies have shown that fractional exhaled nitric oxide (FeNO) levels and bronchodilator responsiveness can vary according to the timing of assessment. However, within conventional daytime testing windows, no significant time‐of‐day differences were observed in the positivity rates of methacholine or mannitol challenge tests. Another large retrospective study likewise reported that the positivity rate for bronchodilator responsiveness decreased as the time of daytime testing was delayed [8, 9]. These findings indicate that asthma‐related diagnostic measures may exhibit temporal dependence; however, bronchodilator responsiveness and inflammatory biomarkers cannot substitute for AHR as directly assessed by bronchial challenge testing. Direct evidence characterizing temporal variation in human AHR remains limited, with current evidence derived primarily from early studies with small sample sizes. Repeated bronchial challenge studies incorporating modern circadian research designs are still lacking. Thus, whether human AHR is independently regulated by the endogenous circadian system, and whether its temporal pattern varies according to the provoking stimulus and clinical phenotype, remain to be determined.

Against this background, this review uses the circadian system as its central biological framework to provide a structured overview of the definition and assessment of time‐dependent AHR, temporal phenotypes observed in humans, potential regulatory mechanisms, and clinical translational implications. Evidence is stratified according to the source of the evidence and the extent to which it directly addresses time‐dependent AHR in humans, with particular emphasis on distinguishing clock‐time variation from endogenous circadian rhythmicity, direct evidence from bronchial challenge testing from supportive evidence based on other measures of airway function, and relatively well‐established mechanistic findings from mechanisms that remain hypothetical and require further validation. With respect to treatment, we further distinguish the effects of ICS administration timing on nocturnal lung function and inflammatory markers supported by randomized trials [10] from other time‐based intervention strategies for which direct evidence regarding human AHR remains limited. This approach delineates the current boundaries of the evidence and highlights future priorities for mechanistic research, clinical assessment, and optimization of treatment timing in time‐dependent AHR.

## 2 Methods

### 2.1 Literature Search Strategy and Study Selection

This review adopted a structured narrative approach to synthesize and critically evaluate clinical and preclinical evidence concerning the time dependence of AHR in asthma, its potential circadian regulatory mechanisms, and its implications for chronotherapeutic strategies. Given the substantial heterogeneity among the available studies, including differences in AHR assessment methods, asthma phenotypes, timing of assessment, and interventions, as well as the inclusion of human interventional studies, controlled physiological studies, observational studies, animal experiments, and ex vivo or in vitro investigations, a quantitative meta‐analysis was not performed. Instead, the evidence was systematically integrated and critically appraised according to its source, directness of relevance to AHR, and degree of certainty of the conclusions.

The databases searched included PubMed/MEDLINE, Web of Science Core Collection, Embase, and Scopus, with coverage from database inception to September 12, 2026. The search strategy comprised five conceptual modules: asthma; AHR and bronchial challenge; circadian rhythms and time; biological mechanisms; and chronotherapy. In addition to core terms such as “asthma,” “airway hyperresponsiveness,” “circadian,” “time of day,” and “chronotherapy,” the AHR module included terms such as “bronchial challenge,” “bronchial provocation,” “airway responsiveness,” “methacholine,” “histamine,” “mannitol,” “adenosine monophosphate,” “hypertonic saline,” “cold air,” “exercise,” “eucapnic voluntary hyperpnea,” “allergen challenge,” “PC_20_,” and “PD_20_.” The circadian module included terms such as “biological night,” “circadian phase,” “chronotype,” “melatonin,” “dim‐light melatonin onset,” “constant routine,” and “forced desynchrony.” Search strategies were adapted for each database using controlled vocabulary, free‐text terms, and database‐specific search operators. The complete PubMed search strategy is provided in Table S1.

Peer‐reviewed studies directly relevant to the core questions of this review were eligible for inclusion, including: (1) studies comparing AHR at different clock times or circadian phases in patients with asthma or relevant populations; (2) human studies evaluating associations between circadian rhythms, sleep, or time‐related factors and airway function, airway inflammation, or clinical outcomes of asthma; (3) animal, ex vivo, or in vitro studies examining the effects of clock components or temporal signals on AHR and related pathways; (4) interventional studies comparing the effects of different medication administration times on AHR, lung function, symptoms, acute exacerbations, or other clinical outcomes; and (5) technical standards, guidelines, and high‐quality reviews used to define concepts related to AHR assessment and circadian rhythms. Studies were excluded if they had no clear relevance to asthma, AHR, or temporal regulation; if they were conference abstracts for which insufficient information was available and a full peer‐reviewed article could not be obtained; or if they merely reproduced previously established conclusions without providing relevant original data. Only full‐text articles published in English were included. Publication year was not used as an exclusion criterion. When recent studies addressed the same question, high‐quality original studies published within the preceding 5 years were preferentially cited, whereas earlier studies were retained when modern alternative evidence was unavailable or when they were necessary to document seminal findings.

After removal of duplicates, the retrieved records were screened sequentially based on their titles, abstracts, and full texts for relevance to the review questions. For included studies, the following information was extracted: study population or experimental model, study design, AHR stimulus and outcome measures, clock time or circadian phase of assessment, control of time‐related confounding factors, intervention characteristics, and major findings. References supporting key conclusions were traced to the original full‐text publications, and additional studies were identified through reference lists and forward citation tracking of key articles and high‐quality reviews. Narrative reviews, systematic reviews, and guidelines were primarily used to define concepts, provide contextual synthesis, and identify relevant original studies. Conclusions regarding treatment efficacy, changes in AHR, and specific mechanistic findings were supported directly by the original studies whenever these were available.

### 2.2 Evidence Classification and Critical Appraisal

Evidence was evaluated along two independent dimensions: the source of the evidence and its directness with respect to the proposed circadian regulation of AHR. Studies from different experimental settings were therefore not arranged into a single linear hierarchy. According to study source, the included evidence was classified into six categories: (I) human interventional studies, including randomized controlled trials, crossover studies, and medication‐timing studies; (II) controlled human physiological studies, including constant‐routine and forced‐desynchrony studies, within‐subject repeated time‐point assessments, and bronchial challenge studies; (III) human observational or epidemiological studies; (IV) in vivo animal studies; (V) ex vivo or in vitro studies; and (VI) indirect evidence derived from other diseases, tissues, or general circadian research. This classification describes the source and study design of the evidence and does not, by itself, indicate its strength.

According to the directness of the evidence for the temporal regulation of AHR, findings were categorized into four groups. Direct human AHR evidence refers to studies in which standardized bronchial challenge testing was repeated at different clock times or circadian phases in patients with asthma, or in which AHR was evaluated as an outcome of medication timing. Supportive human or translational evidence includes human studies that did not repeat bronchial challenge testing but evaluated temporal changes in lung function, airway resistance, symptoms, inflammatory markers, or rescue medication use. Preclinical mechanistic evidence includes animal, ex vivo, or in vitro studies that directly examined the effects of clock components or temporal signals on pathways relevant to AHR. Evidence from other diseases, tissues, or general circadian research was considered indirect evidence and was used only to support biological plausibility or to formulate hypotheses requiring further validation. Studies measuring only lung function, symptoms, or biomarkers were not considered direct evidence of AHR.

For human studies of temporal variation, particular attention was paid to whether within‐subject repeated measurements were used, whether the order of assessments was randomized, whether measurements covered the nighttime period or the full 24 h cycle, whether circadian phase markers such as melatonin were assessed, and whether sleep, posture, physical activity, food intake, environmental exposures, and medication use were controlled. For repeated bronchial challenge studies, the type of provoking stimulus, outcome measures, baseline lung function, interval between assessments, and potential carryover or residual effects were additionally evaluated. For chronotherapy studies, randomization, blinding, crossover sequence, washout periods, adherence, and the clinical relevance of outcomes were further assessed. For animal and in vitro studies, particular attention was given to sampling time, characterization of the light–dark cycle or circadian phase, whether clock pathways were directly manipulated, and the reproducibility of the findings. Across all studies, consistency of results, precision of estimates, and applicability to the clinical asthma population were also considered.

Differences observed between clock times were described only as time‐dependent variation. Findings were attributed to endogenous circadian rhythmicity only when supported by constant‐routine, forced‐desynchrony, or other study designs capable of separating circadian effects from sleep and behavioral cycles, together with appropriate markers of circadian phase. Demonstration that a particular pathway contributes to asthma or AHR was not considered sufficient to establish that the pathway mediates circadian variation in AHR. Accordingly, the main text uses distinct formulations, including “direct human evidence shows,” “supportive human evidence suggests,” “preclinical studies indicate,” and “mechanistic hypothesis,” to avoid presenting preclinical findings or general biological plausibility as mechanisms already established in human asthma. Because this was a narrative review, no standardized numerical scores or pooled evidence grades were assigned across heterogeneous studies.

## 3 Definition and Characteristics of Time‐Dependent AHR

### 3.1 Operational Definition and Assessment of AHR

AHR refers to increased airway sensitivity to stimuli and an exaggerated bronchoconstrictor response and represents an important physiological basis of the variable airflow limitation characteristic of asthma [2]. Clinically, bronchial challenge testing is primarily used to assess patients with recurrent wheeze, cough, chest tightness, or dyspnea in whom conventional pulmonary function testing and bronchodilator testing have not demonstrated variable airflow limitation. A positive challenge test supports the diagnosis of asthma but should be interpreted in conjunction with symptom patterns, baseline lung function, airway inflammation, and response to treatment [1]. Time‐dependent AHR refers to reproducible differences in PC_20_, PD_20_, dose–response slope, or maximal response measured in the same individual at different clock times or circadian phases, while the provoking stimulus, challenge protocol, and outcome measures remain consistent. It therefore reflects temporal variation in the threshold or magnitude of the airway response to a stimulus rather than fluctuations in baseline lung function itself [7]. Bonnet et al. evaluated nine patients with mild asthma and nocturnal symptoms at 4 h intervals, yielding 13 consecutive time points. All participants showed significant and reproducible 24 h variation in histamine‐ or methacholine‐induced PC_20_, whereas significant changes in FEV_1_ were observed in only three patients [4]. Thus, relatively stable baseline FEV_1_ does not exclude temporal variation in AHR, and a nocturnal decline in FEV_1_ cannot substitute for direct assessment of AHR by bronchial challenge testing.

Methacholine is the most widely used provoking agent for assessing direct AHR in clinical practice and human studies. It induces airway contraction primarily by activating muscarinic cholinergic receptors on airway smooth muscle, particularly the M3 receptor, and enables a quantifiable dose–response relationship to be established using progressively increasing inhaled doses [2]. In studies of time‐dependent AHR, the principal value of methacholine challenge testing lies in the use of quantitative measures, such as PC_20_, PD_20_, and dose–response relationships, to compare the airway response threshold and magnitude within the same individual across different clock times or circadian phases, rather than relying solely on a positive or negative result from a single test [6, 11]. PC_20_ and PD_20_ denote the provocative concentration and delivered dose, respectively, required to reduce FEV_1_ by 20% from the baseline value measured at the same assessment [2, 12]. A lower PC_20_ or PD_20_ at night or in the early morning indicates that a smaller amount of stimulus is required to induce the same degree of airway narrowing, consistent with increased airway sensitivity [2, 12].

PC_20_ and PD_20_ primarily characterize the threshold of airway responsiveness and do not indicate how rapidly airway narrowing progresses after the threshold is reached or the maximum degree of bronchoconstriction that can be achieved. Therefore, the dose–response slope and the maximal fall in FEV_1_ or response plateau should also be considered to characterize, respectively, the rate of airway narrowing with increasing stimulus dose and the maximal bronchoconstrictor capacity [2, 11]. Temporal changes in these measures do not necessarily occur in parallel: circadian variation may shift the dose–response curve to the left without substantially altering its overall shape, or may additionally increase the slope or maximal response. Most human studies to date have reported PC_20_, and the available evidence therefore primarily supports increased airway sensitivity at night or in the early morning, while remaining insufficient to determine whether maximal bronchoconstrictor capacity follows the same temporal pattern. To ensure comparability across assessment times, the delivery device, actual delivered dose, inhalation technique, dose intervals, pulmonary function testing procedures, and medication withholding requirements should be kept consistent. Because PC_20_ can be influenced by nebulizer output and inhalation protocols, ERS technical standards recommend preferential use of PD_20_ for comparisons across different assessment times and testing protocols [13].

Indirect challenges include exercise, voluntary hyperventilation with carbon dioxide depletion, cold air, mannitol, hypertonic saline, and adenosine‐based stimuli. These challenges primarily induce airway narrowing through changes in airway surface liquid osmolality, epithelial stress, and the release of endogenous mediators [14, 15, 16]. Because direct and indirect challenges engage distinct physiological pathways, their results should not be considered interchangeable. Oosterhoff et al. performed adenosine 5′‐monophosphate (AMP), methacholine, and propranolol challenge tests at 16:00 and 04:00 in 16 nonsmoking patients with atopic asthma. Among patients with diurnal PEF variability ≥ 15%, the AMP PC_20_ at 04:00 was 2.3 doubling concentrations lower than that at 16:00, whereas the methacholine PC_20_ was 0.9 doubling concentrations lower; the difference between the two responses was statistically significant. Moreover, the temporal variation in the AMP response was strongly correlated with the magnitude of PEF variation (*r* = 0.81, *p* < 0.001) [5]. Baseline FEV_1_ did not differ significantly between the two testing days at the corresponding time points, suggesting that the difference between the two challenge responses could not be explained solely by baseline airway caliber and supporting the possibility that mediator‐dependent indirect responses may exhibit more pronounced temporal variation. However, mast cell activation or the levels of relevant mediators were not measured concurrently in this study; therefore, the specific cellular mechanisms underlying the enhanced nocturnal AMP response cannot be determined from these findings alone.

The assessment of AHR is also influenced by the testing time window, interval between repeated challenges, and a combination of technical and patient‐related factors. Beach et al. compared methacholine PD_20_ at 08:00, 14:00, and 20:00 in 24 patients with asthma and found no significant differences among these daytime time points. However, repeated challenges performed at intervals of no more than 24 h were associated with an increase in PD_20_, suggesting that short‐interval testing may be affected by refractoriness [17]. This negative finding primarily applies to conventional daytime testing windows and does not exclude the possibility of AHR variation during nighttime or early‐morning periods that were not assessed. The nebulizer and its calibration, the actual delivered dose, tidal breathing or deep‐inhalation technique, dose increments, intervals between successive doses, timing of post‐challenge pulmonary function measurements, and the acceptability and reproducibility of FEV_1_ measurements before and after challenge can all affect the estimation of PC_20_, PD_20_, and dose–response curves [18, 19]. The washout period for bronchodilators and anticholinergic agents, ICS treatment, recent infections, allergen or pollutant exposure, exercise, sleep, and body position may also enhance, attenuate, or obscure temporal differences [7]. Age, sex, chronotype, disease severity, and inflammatory phenotype may further influence both the baseline level of AHR and the magnitude of its temporal variation; however, existing repeated‐challenge studies have generally been underpowered for reliable stratified analyses [7, 20, 21, 22]. Therefore, interpretation of time‐dependent AHR should be based on standardized within‐subject comparisons using bronchial challenge testing, with reporting of the challenge stimulus, outcome measures such as PC_20_ or PD_20_, clock time or circadian phase, baseline FEV_1_, pulmonary function test quality, treatment and exposure status, and the interval between repeated challenges (Figure 1).

**FIGURE 1:**
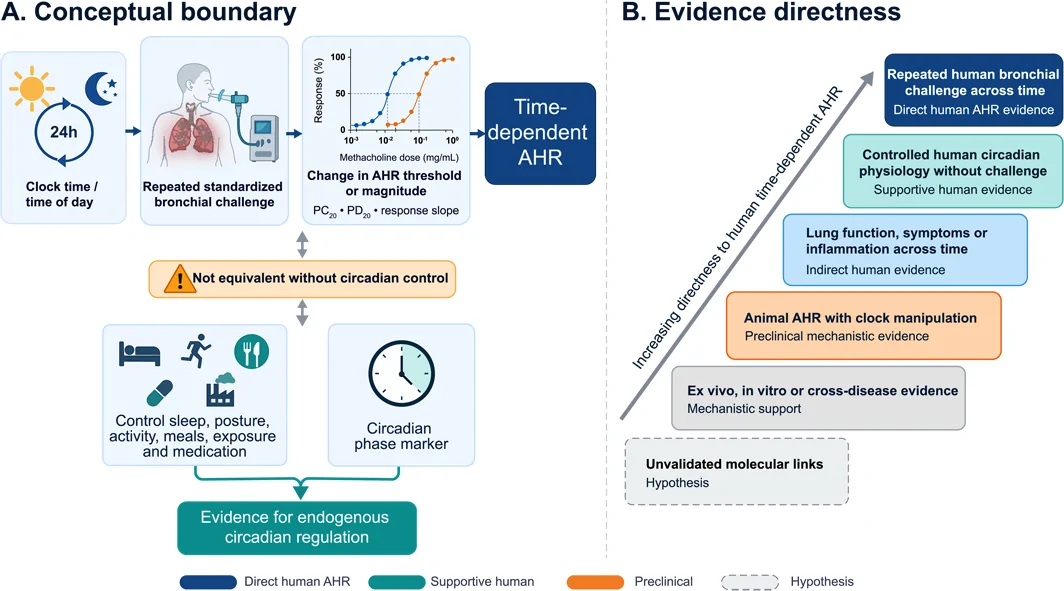
Conceptual boundary and evidence hierarchy for time‐dependent airway hyperresponsiveness. (A) Time‐dependent airway hyperresponsiveness (AHR) is identified by repeating a standardized bronchial challenge at different clock times and demonstrating a change in the response threshold, dose–response slope, or maximal airway narrowing. A clock‐time difference alone does not establish endogenous circadian regulation. Such attribution additionally requires control or systematic distribution of sleep, posture, physical activity, meals, environmental exposures, and medication use, together with assessment of an internal circadian‐phase marker. (B) Hierarchy of evidence according to its directness to human time‐dependent AHR. Repeated bronchial challenge across time provides direct human AHR evidence; controlled human circadian studies without bronchial challenge provide supportive evidence; temporal changes in lung function, symptoms, or inflammation constitute indirect human evidence. Animal studies involving clock manipulation provide preclinical mechanistic evidence, whereas ex vivo, in vitro, and cross‐disease findings offer mechanistic support. Molecular links not validated in relevant experimental models are classified as hypotheses. The ascending arrow represents increasing directness to human time‐dependent AHR rather than overall study quality. Solid dark‐blue, teal, and orange elements denote direct human, supportive human, and preclinical evidence, respectively; dashed gray elements indicate proposed or unconfirmed relationships. AHR, airway hyperresponsiveness; PC_20_, provocative concentration causing a 20% decrease in forced expiratory volume in 1 s; PD_20_, provocative delivered dose causing a 20% decrease in forced expiratory volume in 1 s.

### 3.2 Diurnal Variation in AHR and Endogenous Circadian Rhythmicity: Human Evidence

Circadian rhythms are physiological oscillations generated by the biological clock, with a period of approximately 24 h, and are synchronized by external time cues such as light exposure, food intake, and physical activity [23]. Clock time refers to local social time, whereas circadian phase indicates the position of an individual's endogenous biological clock and can generally be determined using biological markers such as melatonin secretion. Biological nighttime is the rest period defined by endogenous circadian phase and does not necessarily correspond to nighttime according to clock time [24]. Chronotype reflects an individual's preference for the timing of sleep and activity and may result in different biological phases occurring at the same clock time [25]. Therefore, differences in AHR measured at different clock times initially represent an observable time‐dependent phenotype. Only after circadian phase has been established and factors such as sleep, body position, physical activity, food intake, environmental exposures, and medication timing have been controlled can it be further determined whether these variations are independently driven by the endogenous circadian system [7].

Direct human bronchial challenge studies have demonstrated reproducible time‐dependent variation in AHR, although the phase of peak responsiveness varies according to the challenge stimulus. Sly and Landau performed bronchial challenge tests every 6 h in 15 children with asthma and found that histamine responsiveness was highest at 04:00, whereas airway responsiveness induced by hyperventilation with cold, dry air was greatest at 16:00. These differences could not be explained by changes in airway caliber before challenge and persisted despite adequate pharmacological control of symptoms [26]. Van Aalderen et al. compared 9 children with asthma who had nocturnal airflow limitation with 9 who had no apparent nocturnal airflow limitation. Although the decline in FEV_1_ from 16:00 to 04:00 was 21.9% and 2.3% in the two groups, respectively, histamine PC_20_ at 04:00 was lower than that at 16:00 in both groups, with changes of 1.1 and 1.5 doubling doses, respectively. These findings indicate that the increase in nocturnal AHR is not simply an extension of baseline airway narrowing [27]. Mohiuddin and Martin administered the same inhaled allergen challenge in the morning and evening to 10 patients with asthma. A late asthmatic response occurred in 4 patients following morning challenge, compared with 9 following evening challenge. The mean time to onset decreased from 9.4 to 3.1 h, while the maximum decline in FEV_1_ increased from 32.8% to 43.0%. Moreover, methacholine responsiveness 24 h after evening challenge was significantly greater than that observed after morning challenge [28]. Collectively, these studies demonstrate the time dependence of AHR and allergen‐induced airway responses, while also indicating that the airway responses elicited by histamine, cold dry air, and allergen challenge should not be regarded as fully equivalent circadian phenotypes.

Recent human studies that have strictly controlled behavioral and environmental factors have provided further evidence that asthma‐related airway function is regulated by the endogenous circadian system. Scheer et al. used a 38 h constant‐routine protocol and a 196 h forced‐desynchrony protocol, respectively, under dim‐light conditions, while controlling or evenly distributing behavioral factors including sleep–wake cycles, body position, physical activity, and food intake. Both protocols showed that FEV_1_ in patients with asthma reached its lowest level during the biological night, whereas airway resistance was highest, with the nadir occurring at approximately 04:00 in conventional clock time. The probability of symptom‐driven rescue bronchodilator use was approximately fourfold higher during the biological night than during the daytime [7]. These findings demonstrate that the endogenous circadian system can independently regulate baseline airway function and clinical symptoms in asthma and may act in conjunction with sleep and other behavioral factors to further worsen airway status at night. However, bronchial challenge testing was not repeated at different circadian phases in this study. Thus, these findings provide supportive evidence for endogenous circadian regulation of AHR but do not directly establish that AHR itself exhibits an intrinsic endogenous circadian rhythm.

Recent clinical studies further suggest that testing during conventional working hours may not fully capture the temporal variation in AHR. Using data from the RADicA cohort, Wang et al. examined the influence of testing time on asthma diagnostic tests and found that morning testing was more likely to yield higher fractional exhaled nitric oxide levels and a positive bronchodilator response. However, compared with morning testing, the odds ratio for a positive methacholine challenge in the afternoon was 0.88 (95% CI, 0.40–1.98), while that for a positive mannitol challenge was 1.40 (95% CI, 0.48–4.17); neither difference reached statistical significance [8]. Although this study provides recent negative evidence regarding time‐dependent AHR, different participants were assessed at different time points, and testing was largely restricted to routine clinical hours. The early‐morning period in which previous studies suggested peak airway responsiveness was not assessed; therefore, these findings cannot exclude enhanced nocturnal AHR. The SPIRO‐TIMETRY study analyzed bronchodilator responses in 1620 adults and found positive response rates of 28% in the morning and 23% in the afternoon. For each 1 h delay in testing, the adjusted odds of a positive response decreased by 8%, and this association was observed primarily among patients with asthma or suspected asthma [9]. Because bronchodilator responsiveness reflects reversible airflow limitation, it cannot substitute for AHR measured by bronchial challenge. Rather, these findings indicate that the clinical detection of asthma‐related airway dysfunction may itself be time dependent.

It is noteworthy that the evidence directly evaluating temporal variation in human AHR comes predominantly from small‐sample studies conducted in the 1980s and 1990s. This concentration in earlier literature should not simply be regarded as an issue of outdated citations that can be readily replaced by recent reviews or pulmonary function studies; rather, it reflects the fact that the key evidence base in this field has remained insufficiently updated. Although early studies used repeated bronchial challenges in the same individuals at different clock times and provided initial evidence for the time dependence and stimulus specificity of AHR, they were generally limited by small sample sizes, a restricted number of testing time points, study populations predominantly comprising children or patients with nocturnal asthma, inconsistent control of treatment and environmental exposures, and a lack of biomarkers for determining circadian phase. Recent constant‐routine and forced‐desynchrony studies have established that the endogenous circadian system can regulate baseline airway function, but standardized bronchial challenge testing has not yet been incorporated into repeated assessments across different biological phases. Thus, current evidence supports clock‐time‐dependent variation in AHR in some patients with asthma, but remains insufficient to determine its peak phase, rhythm amplitude, stimulus specificity, or differences in clinical phenotype, and does not yet establish that the endogenous circadian clock independently regulates human AHR. Future studies should adopt within‐subject randomized crossover designs covering both the biological night and daytime, incorporate circadian phase markers such as melatonin, and control for sleep, body position, medication timing, and environmental exposures. Separate standardized challenge protocols for direct and indirect stimuli should also be implemented with sufficiently long intervals between repeated challenges to minimize refractoriness or residual effects of prior challenges (Figure 1, Table 1).

## 4 Potential Mechanisms Underlying Circadian Regulation of AHR

### 4.1 Neuroendocrine and Autonomic Nervous System Gating

Airway caliber and the threshold for bronchoconstriction are not constant but are dynamically regulated by neuroendocrine and autonomic circadian signals. Clinically, nocturnal and early‐morning worsening of AHR has been clearly observed in some patients with asthma; however, the mechanisms underlying this diurnal variation remain incompletely understood. Given the central role of the central clock in circadian regulation, establishing a neuroendocrine link between the circadian system and AHR may help explain, at least in part, the temporal characteristics of AHR. However, high‐quality evidence in this area is currently derived predominantly from animal models and ex vivo studies and therefore remains at the preclinical stage.

First, with respect to the autonomic nervous system, particularly the parasympathetic pathway, an early human study demonstrated that airway responsiveness to histamine and methacholine in patients with mild asthma who experienced nocturnal awakenings due to dyspnea exhibited a stable circadian pattern, with greater responsiveness during the night and early morning; this variation was not fully synchronized with fluctuations in baseline pulmonary function [4]. Consistent with these findings, two small‐sample human clinical studies conducted by Morrison and colleagues indirectly indicated that parasympathetic, or vagal, activity may increase to some extent during the night in patients with asthma [29, 30]. Subsequent studies have provided mechanistic insights into a potential chain linking nocturnal parasympathetic activity with increased airway responsiveness. A review by Kistemaker et al. [31] proposed that acetylcholine may serve as a bridging mediator between parasympathetic activation and AHR, with the M3 muscarinic receptor playing a key role in mediating this effect. In addition to M3 receptors, another study showed that M1 receptors located in the alveolar wall may further enhance cholinergic reflexes by facilitating neurotransmitter transmission within parasympathetic ganglia [32]. Taken together, these findings suggest that increased nocturnal airway responsiveness in patients with asthma may be closely associated with enhanced vagal activity. Nevertheless, the small sample sizes and limited temporal and generalizability of previous human studies warrant caution in extrapolating these findings. Further prospective studies with larger cohorts, more direct assessments, and appropriate ethical and safety considerations are needed to validate the circadian relationship between parasympathetic activity and airway responsiveness.

However, these clinical observations alone cannot determine the relative contributions of altered neural input and intrinsic rhythmic regulation within the airway tissues to this time‐dependent phenomenon. Recent animal studies have provided important evidence for the latter. In a house dust mite (HDM)‐induced model of allergic airway inflammation, AHR exhibited marked time dependence, and this difference was preserved in precision‐cut lung slices ex vivo, indicating the presence of local regulatory pathways within the airway tissues that maintain this temporal pattern. These findings provide key experimental evidence that the local pulmonary clock may contribute to circadian regulation of AHR [33]. Further studies showed that loss of REV‐ERB*α* abolished the diurnal variation in AHR and was accompanied by loss of rhythmic expression of Chrm1 and Chrm3, particularly Chrm3. Consistently, methacholine‐induced contraction of ex vivo lung slices no longer exhibited a clear time‐dependent pattern [33]. These findings suggest that REV‐ERB*α* may regulate the time‐dependent contractile response of the airways to cholinergic stimulation by controlling the rhythmic expression of muscarinic receptors, thereby providing a potential mechanism through which the local pulmonary clock gates AHR according to circadian time. Nevertheless, it should be emphasized that the REV‐ERB*α*–cholinergic receptor–AHR pathway has so far been established primarily in animal and ex vivo models, and its complete causal relationship in human asthma remains to be determined.

At the neuroendocrine level, endogenous cortisol (a glucocorticoid) released rhythmically through the hypothalamic–pituitary–adrenal (HPA) axis serves as an important entraining signal for peripheral circadian rhythms [34, 35] and a key signaling molecule in the regulation of airway inflammation [36]. Human studies have established that serum or plasma cortisol exhibits a pronounced circadian rhythm, reaching a nadir during the night after sleep onset and beginning to rise before early morning [37, 38]. This temporal pattern partially overlaps with the phase during which asthma symptoms, airway obstruction, and AHR worsen. Given the anti‐inflammatory effects of cortisol, particularly those mediated by glucocorticoids, early studies proposed that the nocturnal cortisol nadir and the resulting reduction in endogenous anti‐inflammatory activity might be an important contributor to nocturnal asthma [39]. However, this temporal concordance alone does not establish a causal relationship. Indeed, a classic human intervention study suggested that the circulating cortisol rhythm may be insufficient to explain the pathophysiology of nocturnal asthma: even when exogenous cortisol was infused at night to prevent the decline in circulating glucocorticoid levels, the nocturnal reduction in peak expiratory flow rate (PEFR) was not effectively reversed in most patients [40]. This finding suggests that the circadian variation in cortisol‐related AHR may not be attributable solely to changes in hormone abundance; how pulmonary tissues respond to rhythmic hormonal signals is also likely to be an important consideration.

The biological effects of glucocorticoids depend not only on their circulating concentrations but are also subject to time‐dependent regulation by the intrinsic clock of target tissues and the function of the glucocorticoid receptor (GR) [41, 42]. A recent animal study using a model of asthma showed that Brain and Muscle Arnt‐like 1(BMAL1) deficiency not only exacerbated airway inflammation but also impaired the anti‐inflammatory efficacy of glucocorticoids and induced glucocorticoid resistance. A series of experiments further identified the DUSP4–p38 mitogen‐activated protein kinase (p38 MAPK) pathway as a key mechanistic link connecting BMAL1 to glucocorticoid resistance and airway inflammation. BMAL1 was shown to regulate GR phosphorylation and activation through this pathway, whereas pharmacological inhibition of DUSP4 effectively restored glucocorticoid sensitivity. These findings provide new mechanistic insight into circadian disruption‐associated glucocorticoid resistance in asthma and identify a potential therapeutic target [41]. Regarding GR‐mediated glucocorticoid responses, an earlier clinical study found that patients with nocturnal asthma exhibited lower GR binding affinity during the early morning compared with patients without nocturnal asthma and non‐asthmatic controls. The anti‐inflammatory effects of glucocorticoids were correspondingly attenuated, suggesting that worsening nocturnal airway inflammation may be associated with a localized or cellular state of reduced glucocorticoid responsiveness [42]. Thus, nocturnal asthma may reflect the combined effects of circulating hormonal rhythms and the local pulmonary clock. Although human studies have indicated phase‐dependent variation in glucocorticoid responsiveness in nocturnal asthma, and animal studies have begun to elucidate its underlying molecular basis, studies directly linking cortisol rhythms, the pulmonary clock, and the temporal dynamics of AHR remain limited.

In addition to glucocorticoids, melatonin is a prototypical nocturnal endocrine signal, with a well‐established temporal pattern characterized by a marked increase in plasma levels at night [43]. Regarding its association with asthma, an early clinical study found that patients with nocturnal asthma had a higher melatonin peak and a delayed peak phase compared with healthy controls, while pulmonary function parameters were negatively correlated with melatonin levels, suggesting a potential link between endogenous melatonin levels and nocturnal deterioration of airway function [44]. With respect to its underlying mechanisms, existing non‐human studies indicate that melatonin may exert bidirectional effects on airway responsiveness through multiple pathways, including immune inflammation, airway smooth muscle contraction, and redox homeostasis. An early in vitro study demonstrated that pharmacological concentrations of melatonin could inhibit cyclic adenosine monophosphate (cAMP) signaling through the MT_2_ receptor and enhance calcium mobilization, thereby promoting contraction of human airway smooth muscle [45]. Although this finding provides some support for a potential pro‐contractile effect of melatonin, results obtained at pharmacological concentrations cannot readily be extrapolated to the physiological effects of nocturnal increases in endogenous melatonin on airway tone. In contrast, a study using an ozone‐aggravated allergic asthma model demonstrated antioxidant and antiasthmatic effects of melatonin, which alleviated oxidative stress, airway inflammation, and AHR by targeting transient receptor potential vanilloid 1 (TRPV1) and activating the nuclear factor erythroid 2‐related factor 2 (Nrf2) antioxidant pathway [46]. Similarly, a recent study in mice with allergic airway inflammation further suggested that melatonin can reprogram the redox metabolism of group 2 innate lymphoid cells (ILC2s), enhance NRF2‐mediated glutathione synthesis, and suppress type 2 inflammation and AHR; this mechanism was also partially validated in primary human ILC2s [47]. Thus, the effects of melatonin on asthma‐related airway phenotypes are not simply unidirectional, either pro‐inflammatory or anti‐inflammatory, but rather appear to be complex and context dependent. Although human studies have documented alterations in the endogenous melatonin rhythm in patients with nocturnal asthma [44], direct human phase‐specific intervention studies demonstrating that altered melatonin rhythmicity itself is a primary driver of the circadian variation in AHR are lacking. In previous clinical trials, exogenous melatonin supplementation improved sleep quality but did not significantly improve asthma symptoms, PEFR, or rescue medication use [48]. Therefore, compared with the autonomic cholinergic and glucocorticoid axes, melatonin is currently better regarded as a potentially modulatory nocturnal signal and candidate regulator rather than an established core mechanism governing the circadian gating of AHR. Future studies combining manipulation of endogenous melatonin rhythmicity, receptor‐specific interventions, and circadian phase‐specific experimental designs are needed to clarify its causal role and temporal dependence in nocturnal asthma (Figure 2).

**FIGURE 2:**
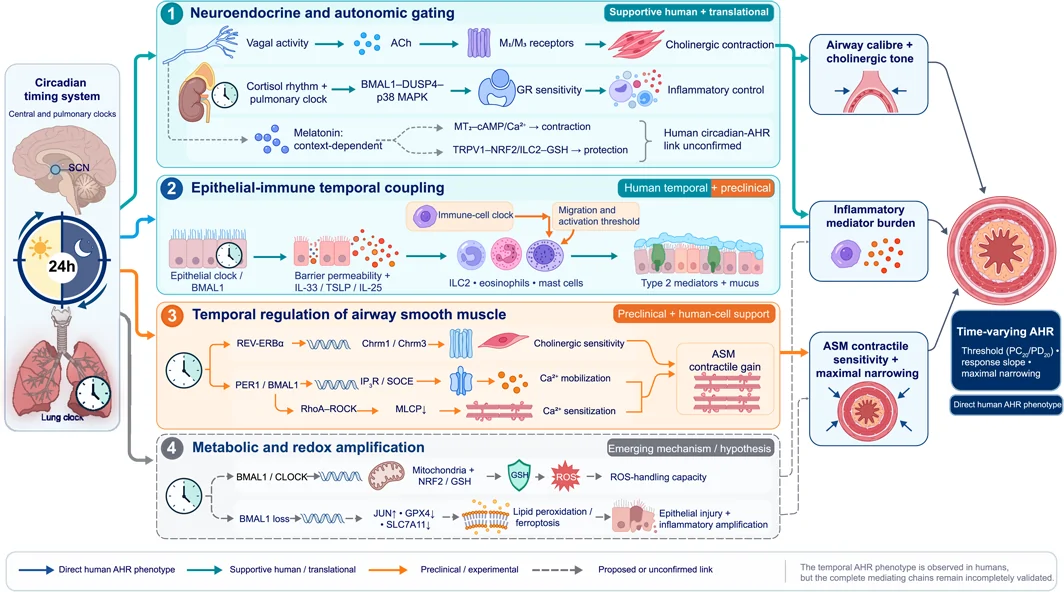
Evidence‐stratified mechanisms potentially contributing to time‐varying airway hyperresponsiveness in asthma. Central and pulmonary clocks may temporally gate airway responsiveness through four interacting domains. (1) Neuroendocrine and autonomic pathways regulate vagal acetylcholine release, muscarinic‐receptor signaling, cortisol‐dependent glucocorticoid sensitivity, and context‐dependent melatonin effects. (2) Epithelial and immune clocks may alter barrier permeability, epithelial alarmin release, immune‐cell migration and activation, type 2 inflammation, and mucus production. (3) Airway smooth‐muscle clocks may influence cholinergic sensitivity, intracellular Ca^2+^ mobilization, Ca^2+^ sensitization, and contractile gain through REV‐ERB*α*–Chrm1/Chrm3, PER1/BMAL1–IP_3_R/SOCE, and RhoA–ROCK–MLCP pathways. (4) Clock‐related metabolic and redox regulation may modify mitochondrial function, antioxidant capacity, lipid peroxidation, ferroptosis, epithelial injury, and inflammatory amplification. These processes converge on airway caliber and cholinergic tone, inflammatory‐mediator burden, and airway smooth‐muscle contractility, which may produce temporal variation in challenge threshold, response slope, and maximal airway narrowing. Dark‐blue arrows indicate the observed human time‐dependent AHR phenotype; teal arrows indicate supportive human or translational evidence; orange arrows indicate preclinical or experimental evidence; dashed gray arrows indicate proposed or unconfirmed links. The human temporal AHR phenotype has been observed, but most molecular pathways and their endogenous circadian mediation remain incompletely validated in patients. ACh, acetylcholine. AHR, airway hyperresponsiveness; ASM, airway smooth muscle; BMAL1, brain and muscle ARNT‐like protein 1; cAMP, cyclic adenosine monophosphate; Chrm1/Chrm3, cholinergic receptor muscarinic 1 and 3, respectively; DUSP4, dual‐specificity phosphatase 4; GPX4, glutathione peroxidase 4; GR, glucocorticoid receptor; GSH, glutathione; IL, interleukin; ILC2, group 2 innate lymphoid cell; IP3R, inositol 1,4,5‐trisphosphate receptor; MLCP, myosin light‐chain phosphatase; MT2, melatonin receptor 2; NRF2, nuclear factor erythroid 2‐related factor 2; PC_20_, provocative concentration causing a 20% decrease in forced expiratory volume in 1 s; PD_20_, provocative delivered dose causing a 20% decrease in forced expiratory volume in 1 s; PER1, period circadian regulator 1; REV‐ERB*α*, nuclear receptor subfamily 1 group D member 1; RhoA, Ras homolog family member A; ROCK, Rho‐associated protein kinase; ROS, reactive oxygen species; SCN, suprachiasmatic nucleus; SLC7A11, solute carrier family 7 member 11; SOCE, store‐operated Ca^2+^ entry; TRPV1, transient receptor potential vanilloid 1; TSLP, thymic stromal lymphopoietin.

### 4.2 Epithelial–Immune Temporal Coupling

The airway epithelium is not merely a physical barrier but an important interface linking environmental stimuli, innate immunity, and airway effector responses. Ex vivo studies have demonstrated distinct molecular circadian rhythms in primary human airway epithelial cells. In contrast, airway epithelial cells from patients with asthma exhibit altered rhythmic amplitude and phase of several core clock genes as well as inflammatory and chemokine genes, indicating that the airway epithelium possesses detectable temporal biological characteristics [49]. However, sufficient longitudinal human data are still lacking to establish that these epithelial rhythmic alterations directly mediate the diurnal variation in AHR. A more plausible interpretation is therefore that the airway epithelium may provide different local environments for airway inflammation and responsiveness at different times of day by modulating barrier integrity, alarmin release, and immune‐cell recruitment. Whether these epithelial changes ultimately determine the diurnal variation in AHR remains to be established in longitudinal human studies.

Recent animal studies provide relatively direct clues supporting this concept. In a chronic HDM‐induced model of allergic airway inflammation, selective deletion of Bmal1 in airway Club cells abolished the normal diurnal rhythms of airway permeability, pulmonary eosinophil accumulation, and Th2‐cell recruitment [50]. These findings suggest that the intrinsic pulmonary clock may regulate the temporal window for immune‐cell entry into the airways by modulating epithelial barrier function. However, it should be emphasized that this evidence is derived primarily from animal models, with barrier integrity and inflammatory‐cell recruitment, rather than AHR itself, as the principal endpoints. Thus, these findings do not establish the epithelial clock as a determinant of the circadian rhythm of AHR.

The epithelial clock–immune recruitment axis may ultimately converge on AHR through multiple pathways, among which alarmin signaling is particularly noteworthy. Airway epithelial‐derived alarmins, including interleukin‐33 (IL‐33), thymic stromal lymphopoietin (TSLP), and interleukin‐25 (IL‐25), can act on ILC2s, dendritic cells, T cells, and eosinophils to promote type 2 inflammation, mucus secretion, and AHR [51, 52]. Notably, the association between abnormal epithelial alarmin signaling and AHR is supported by human evidence. A 2024 study showed that sputum TSLP levels were significantly associated with mannitol‐induced AHR in patients with asthma, independently of eosinophil levels and FeNO, suggesting that epithelial‐derived signals may not simply reflect airway inflammation but may also contribute to the development of an AHR‐prone state [53]. Similarly, previous clinical studies have shown that higher epithelial IL‐25 expression is associated with greater airway hyperresponsiveness and a type 2 inflammatory phenotype [54]. However, these studies primarily addressed whether alarmins are associated with AHR, rather than whether alarmins drive AHR in a circadian manner. To date, only limited ex vivo evidence suggests that reduced BMAL1 expression in severe asthma is accompanied by increased levels of alarmins such as IL‐25, IL‐33, and TSLP [41]. Therefore, alarmins are currently better regarded as potentially time‐sensitive nodes linking the epithelial clock to airway inflammatory responsiveness, rather than directly extrapolating the established involvement of alarmins in AHR to a role in driving the circadian rhythm of AHR.

Further, the temporal window shaped by epithelial‐derived signals may also be modulated by intrinsic temporal programs within immune cells. Human studies have shown pronounced diurnal variations in the numbers of total leukocytes and eosinophils in induced sputum from patients with mild asthma. Eosinophil‐derived neurotoxin (EDN) levels were significantly elevated at 7:00 a.m., and this variation was associated with rhythmic changes in lung function [55]. These findings suggest that the recruitment and activation of airway immune cells are themselves subject to marked temporal regulation. Consistent with this notion, recent evidence has shown altered expression patterns of core clock proteins in peripheral blood eosinophils from patients with asthma [56]. However, it should be noted that this study examined eosinophils isolated from peripheral blood rather than cells directly involved in local airway inflammation, such as those obtained from sputum or bronchoalveolar lavage. Thus, these findings are better regarded as a potential indication that intrinsic immune‐cell clocks may contribute to the temporal regulation of asthma‐related processes, rather than as direct evidence of circadian immune activity within the airways. In other words, epithelial‐derived signals and intrinsic immune‐cell clocks may jointly shape the temporal dynamics of airway inflammation at two complementary levels: the “local microenvironment” and the “cellular response threshold,” respectively.

Taken together, the available evidence supports a potential “two‐layer temporal coupling” between the airway epithelium and immune cells. The epithelial clock may define the temporal window for immune recruitment by regulating barrier function, alarmin release, and chemotactic signals, whereas intrinsic clocks within immune cells may further determine the time‐dependent sensitivity of their migration, activation, and effector responses. When these two temporal programs converge at specific phases of the circadian cycle, they may amplify local inflammatory signaling and increase susceptibility to AHR. Although direct human evidence establishing a complete causal chain from the “epithelial clock–immune recruitment–AHR” axis remains limited, current evidence from both animal models and human studies provides multilayered support for this working model (Figure 2).

### 4.3 Temporal Regulation of Airway Smooth Muscle Reactivity

Airway smooth muscle (ASM) is the principal effector tissue directly responsible for AHR. Thus, in addition to upstream neural and inflammatory signals, the intrinsic temporal dependence of ASM contractile responses may contribute to why the same external stimulus produces different degrees of airway narrowing at different biological phases. However, it is important to emphasize that current human studies have primarily established the involvement of ASM in AHR and its contractile phenotype, whereas there is insufficient evidence that ASM in adults with asthma exhibits intrinsic circadian oscillations synchronized with AHR. Accordingly, relevant mechanistic clues currently derive mainly from animal models and ex vivo tissue studies.

The most direct evidence to date comes from studies of REV‐ERB*α*. As described above, the animal study by Durrington et al. [33] not only demonstrated that the temporal dependence of HDM‐induced AHR in mice with allergic airway inflammation was retained in precision‐cut lung slices ex vivo, but also showed that canonical genes associated with the contractile apparatus, including Acta, Mylk1, and sm‐mhc, did not exhibit pronounced temporal variation. In contrast, Chrm1 and Chrm3 expression displayed time‐dependent patterns that were influenced by REV‐ERB*α* status. These findings suggest that circadian regulation of ASM may not simply involve periodic changes in the expression of the contractile apparatus as a whole, but may instead act at the level of stimulus sensing and downstream signal amplification. Nevertheless, this interpretation is based primarily on animal models and ex vivo lung tissue and therefore cannot yet be directly extrapolated to human asthma.

Human ASM studies provide additional cellular evidence supporting this possibility. Human airway smooth muscle cells express core clock components, including Period circadian protein homolog 1 (PER1), (Period circadian protein homolog 2) PER2, Cryptochrome‐1 (CRY1), Circadian locomoter output cycles protein kaput (CLOCK), and BMAL1. Manipulation of PER1 or BMAL1 altered store‐operated calcium entry (SOCE) and histamine‐induced intracellular Ca^2+^ responses, whereas restoration of clock protein expression partially corrected hyperoxia‐induced abnormalities in Ca^2+^ homeostasis [57]. These findings indicate that ASM possesses intrinsic temporal biological properties and can couple clock status to Ca^2+^ homeostasis. However, the study used developing human ASM and a hyperoxia model, with a primary focus on airway injury associated with oxygen exposure rather than circadian AHR in adult asthma. Therefore, its findings are more appropriately interpreted as mechanistic support at the human cellular level rather than as direct evidence of circadian AHR regulation in asthma.

The above findings suggest a potential coupling between the intrinsic clock of ASM and Ca^2+^ homeostasis. Given that Ca^2+^ is a central mediator of ASM contraction, together with evidence that Ca^2+^ signaling itself exhibits temporal dependence [58], Ca^2+^ may represent an important candidate node linking the ASM clock to contractile responses. Ca^2+^‐dependent regulation of ASM contraction primarily involves two mechanisms: Ca^2+^ mobilization mediated by inositol 1,4,5‐trisphosphate receptors (IP_3_Rs) and SOCE, and Ca^2+^ sensitization of the contractile apparatus through pathways such as RhoA–ROCK, which inhibit myosin light chain phosphatase (MLCP) and sustain myosin light chain phosphorylation [59, 60, 61]. Mechanistically, therefore, if the circadian clock alters the state of Ca^2+^ mobilization or Ca^2+^ sensitization, ASM could theoretically exhibit different contractile response gains across biological phases. However, there is currently no direct evidence that these Ca^2+^‐regulatory processes themselves exhibit circadian rhythms synchronized with AHR. Thus, these mechanisms are better considered plausible explanations for clock‐dependent modulation of ASM reactivity rather than established pathways underlying the diurnal variation in AHR.

Taken together, the available evidence supports a model in which the intrinsic pulmonary clock may regulate contractile receptors and Ca^2+^ handling, thereby conferring phase‐dependent differences in the contractile threshold or signal amplification capacity of ASM. When these intrinsic differences coincide with external bronchoconstrictor stimuli, they may further modulate the magnitude of AHR [33, 57]. At present, direct evidence supporting this model comes predominantly from animal and ex vivo studies, whereas human evidence remains limited to the demonstration that the ASM clock and contraction‐related signaling can be modulated by temporal regulatory mechanisms. Thus, ASM may represent a potential terminal effector layer linking the intrinsic pulmonary clock to the AHR phenotype, although the specific molecular nodes involved and their phase relationships with upstream temporal signals remain to be determined (Figure 2).

### 4.4 Metabolic and Redox Regulation: An Emerging Mechanism

Beyond neuroendocrine regulation, epithelial–immune interactions, and ASM reactivity, circadian control of cellular metabolism and redox homeostasis may represent another layer through which biological timing influences AHR. Compared with the mechanisms discussed above, however, direct evidence linking the circadian clock, redox regulation, and AHR remains limited. This pathway is therefore better regarded as an emerging amplification mechanism that may connect clock status with airway inflammation and effector responses.

Oxidative and antioxidant processes, which together constitute a critical component of cellular energy production and reactive oxygen species (ROS) metabolism, are jointly regulated by the circadian clock, nutritional status, and neuroendocrine signals. A growing body of evidence indicates that the core clock factors BMAL1 and CLOCK can influence mitochondrial dynamics and metabolism [62], as well as Nrf2‐dependent antioxidant defense pathways, including glutathione metabolism [63], thereby generating time‐dependent changes in cellular energy production and ROS clearance. Thus, circadian regulation may affect not only how much ROS is produced, but also the capacity of cells to cope with oxidative stress during different temporal windows.

Consistent with this concept, a recent database‐based study identified abnormalities in mitochondrial structure and function in the airway epithelium of patients with asthma and in animal models [64]. Other studies have further shown that mitochondrial ROS are not merely a consequence of oxidative injury but can also act as signaling molecules that promote inflammatory responses [65]. Given that the airway epithelium is itself susceptible to oxidative stress, excessive ROS may further disrupt epithelial barrier integrity, alter inflammatory signaling, and contribute to airway remodeling and AHR [66]. Accordingly, if circadian regulation shifts mitochondrial metabolism or antioxidant capacity during specific temporal windows, the same magnitude of inflammatory or environmental challenge could generate different levels of oxidative stress. This temporal mismatch in redox burden may represent an emerging mechanism contributing to increased nocturnal susceptibility to AHR. Nevertheless, although substantial experimental evidence supports clock–redox coupling in lung tissue and various cellular models [67, 68], direct evidence that this coupling generates a 24 h redox oscillation synchronized with AHR in the human asthmatic airway remains lacking.

Notably, recent findings have begun to provide asthma‐specific mechanistic evidence linking circadian disruption to airway oxidative injury. A 2026 study reported that BMAL1 deficiency in a HDM‐induced asthma model markedly increased airway epithelial ROS and lipid peroxidation, accompanied by reduced expression of glutathione peroxidase 4 (GPX4) and solute carrier family 7 member 11 (SLC7A11). Pharmacological inhibition of ferroptosis partially reversed the exacerbated airway inflammation and remodeling associated with BMAL1 deficiency. Moreover, BMAL1 deficiency similarly enhanced HDM‐induced lipid peroxidation and ROS accumulation in human bronchial epithelial cells [69]. These findings link the core circadian clock to airway oxidative injury, lipid peroxidation, and ferroptosis, providing a new mechanistic clue as to how circadian disruption may increase airway susceptibility to inflammatory stimuli.

However, it is important to distinguish between circadian disruption promoting oxidative injury and oxidative stress mediating the diurnal variation in AHR. Human studies have yet to directly compare airway mitochondrial ROS levels, antioxidant capacity, and AHR across circadian phases under rigorously controlled conditions, and causal evidence from phase‐specific interventions remains limited. A more plausible mechanistic framework at this stage is that the circadian clock coordinates mitochondrial metabolism and antioxidant defenses, thereby altering the oxidative stress threshold of airway cells across different temporal windows [62, 63]. When asthmatic inflammation or exogenous oxidative challenges coincide with such a time‐dependent vulnerability window, increased ROS and lipid peroxidation may further amplify epithelial injury, inflammatory signaling, and ASM reactivity, ultimately enhancing AHR [65, 66]. This model is biologically plausible, but its role in the circadian variation of AHR remains primarily mechanistic and requires further validation.

Accordingly, metabolic and redox mechanisms are better viewed as a potential amplification layer underlying the diurnal variation in AHR rather than as an independent core driver. Future studies should simultaneously assess mitochondrial function, ROS and lipid peroxidation, and AHR across different circadian phases in patients with asthma while rigorously controlling for potential confounders, including sleep, physical activity, dietary intake, and medication timing. Phase‐specific manipulation of clock components or redox pathways will also be important for establishing the causal contribution of this mechanism to circadian variation in AHR in human asthma (Figure 2).

## 5 Chronotherapy and Clinical Translation

### 5.1 Timing of Inhaled Corticosteroid Administration

Among current chronotherapy studies in asthma, ICS have the most direct clinical evidence in humans. The chronopharmacological rationale is not simply to shift ICS administration from daytime to nighttime, but rather to align glucocorticoid exposure with temporal windows characterized by increased nocturnal airway inflammation, airflow limitation, and AHR. Early clinical studies suggested that the efficacy of ICS may be substantially time dependent. In a randomized clinical trial, Pincus et al. [70] compared triamcinolone acetonide 800 μg administered once daily at 3:00 p.m. with 200 μg administered four times daily for 4 weeks. The two regimens produced broadly comparable effects on FEV_1_, morning and evening PEF, *β*_2_‐agonist use, and systemic effects, suggesting that once‐daily afternoon administration could achieve efficacy comparable to that of conventional multiple‐dose treatment. The same group subsequently conducted a randomized comparison of administration at 8:00 a.m., 5:30 p.m., or four times daily. The 5:30 p.m. regimen produced greater improvements in morning and evening PEF and other measures than the 8:00 a.m. regimen, while achieving overall efficacy similar to that of four‐times‐daily administration; no significant differences in systemic effects were observed among the three regimens [71]. These early human studies thus provided initial clinical evidence that the timing of ICS administration itself may influence therapeutic effects.

Subsequent studies comparing morning and evening administration of different ICS, including ciclesonide and fluticasone furoate, have yielded more heterogeneous results. For example, in 209 patients with asthma, Postma et al. [72] found that morning and evening administration of ciclesonide produced broadly comparable improvements in most lung function measures, symptoms, and rescue medication use, although evening administration resulted in greater improvement in morning PEF. For fluticasone furoate, Woodcock et al. [73] reported that once‐daily evening administration of 400 μg produced a pre‐dose evening FEV_1_ response comparable to that achieved with 200 μg twice daily, whereas morning administration produced a relatively smaller improvement in the corresponding morning measure. However, subsequent studies directly comparing morning and evening administration have generally failed to demonstrate a clear clinically meaningful difference in overall efficacy [74]. Similarly, randomized crossover studies of fluticasone furoate/vilanterol combination therapy and fluticasone furoate monotherapy showed broadly comparable improvements in 24 h weighted mean FEV_1_ and other pulmonary function measures with morning versus evening administration [75, 76].

Taken together, the current clinical evidence supports some degree of temporal dependence and heterogeneity in ICS efficacy across patients and formulations, rather than a fixed optimal administration phase applicable to all ICS. The potential value of chronotherapy may therefore lie in optimizing drug exposure according to the individual's diurnal pattern of disease activity, rather than simply shifting routine ICS administration to nighttime. Such clinical heterogeneity may partly reflect differences in endogenous circadian timing and phase‐dependent glucocorticoid sensitivity in patients with asthma. In an overnight human study by Krakowiak et al. [77], 10 patients with mild‐to‐moderate asthma and 10 healthy controls underwent blood sampling every 6 h. The study showed altered peripheral molecular clock rhythms in patients with asthma, and peripheral blood mononuclear cells exhibited significantly greater responses to immune stimulation and glucocorticoid‐mediated suppression at 4:00 p.m. than at 4:00 a.m. Although this study investigated human circadian biology rather than drug efficacy, it provides important mechanistic support for the possibility that afternoon administration may confer greater anti‐inflammatory effects in some patients with asthma.

Of particular interest, a randomized three‐period crossover trial published in *Thorax* in 2025 provided some of the most direct clinical evidence to date supporting ICS chronotherapy. The study compared different administration timings of beclometasone dipropionate in patients with mild‐to‐moderate atopic asthma and found that all three regimens improved asthma control and reduced FeNO. However, afternoon once‐daily administration produced the most pronounced improvement in nocturnal airflow limitation, particularly by attenuating the decline in FEV_1_ measured at 10:00 p.m. Afternoon administration was also associated with a greater suppression of the nocturnal and early‐morning rise in blood eosinophils, whereas the different dosing regimens did not significantly affect serum cortisol levels [10]. These findings suggest that optimizing the timing of ICS administration may not simply involve shifting treatment toward nighttime. Rather, administering the drug in the afternoon may allow its anti‐inflammatory effects to better cover the subsequent period of nocturnal deterioration in airway function and inflammatory activity, providing new clinical support for individualized ICS chronotherapy based on the diurnal pattern of disease activity [10].

Overall, current clinical evidence supports a potential therapeutic relevance of ICS administration timing, but the magnitude and consistency of this benefit are not yet sufficient to warrant changes to conventional dosing principles. A 2018 systematic review and meta‐analysis found that once‐daily evening administration produced a modest improvement in lung function compared with morning administration; however, substantial methodological and clinical heterogeneity across the included studies limits the interpretation of this finding [78]. Together with the more recent randomized crossover evidence suggesting potential benefits of afternoon administration, these data support viewing ICS dosing time as a potential individualized treatment variable rather than as a fixed dosing principle applicable to all patients. From a clinical research perspective, patients with prominent nocturnal or early‐morning symptoms, marked diurnal variation in lung function, or a greater nocturnal inflammatory burden may represent particularly relevant populations in whom the potential benefits of chronotherapy warrant further evaluation. Future large‐scale, long‐term randomized clinical trials incorporating chronotype, circadian phase, asthma phenotype, and specific ICS characteristics will be needed to define the most appropriate administration timing and target populations, thereby advancing ICS chronotherapy from empirical adjustment toward more individualized application (Table 1).

### 5.2 Timing of Other Existing Asthma Therapies

Compared with ICS, evidence supporting chronotherapy for other asthma controller medications is substantially more limited, and studies have not yet established a consistent “optimal dosing phase.” Available evidence has focused primarily on long‐acting bronchodilators, leukotriene receptor antagonists, and systemic corticosteroids, whereas chronopharmacological studies of biologics remain at an early stage.

For long‐acting muscarinic antagonists (LAMAs) and long‐acting *β*_2_‐agonists (LABAs), existing studies have primarily examined dosing frequency and 24 h pharmacodynamic coverage rather than directly comparing morning and evening administration. For example, a randomized crossover study of tiotropium compared 5 μg once‐daily evening administration with 2.5 μg twice‐daily administration in the morning and evening. Both regimens provided sustained bronchodilation over 24 h and improved 0–24 h area under the curve for FEV_1_ (FEV_1_ AUC_0_–_24_ h), peak and trough FEV_1_, and morning and evening PEF compared with placebo, with no significant difference between the two dosing regimens [79]. These findings suggest that, for LAMAs, timing optimization currently relates primarily to maintaining continuous bronchodilator coverage throughout the day rather than to a clearly established phase‐dependent therapeutic effect. Similarly, studies of LABAs have mainly demonstrated that their prolonged bronchodilator effects can cover nocturnal and early‐morning airflow limitation, without establishing a consistent morning or evening advantage independent of dose and dosing frequency [80]. Thus, although the nocturnal increase in vagal tone and enhanced airway smooth muscle reactivity provide a physiological rationale for considering the timing of LABA/LAMA administration, clinical studies to date have focused predominantly on pulmonary function and 24 h bronchodilator effects rather than direct AHR outcomes. Current evidence therefore remains insufficient to support routine adjustment of LABA/LAMA administration timing according to circadian patterns.

Compared with LABAs/LAMAs, leukotriene receptor antagonists (LTRAs) have been investigated more directly in relation to dosing time, although these studies have likewise failed to demonstrate a clear and consistent advantage of evening over morning administration. For example, Pajaron‐Fernandez et al. [81] used a randomized crossover design to compare morning and evening administration of montelukast in children with exercise‐induced bronchoconstriction. Both dosing schedules significantly reduced the post‐exercise decline in FEV_1_ and the bronchoconstrictive response, with no significant difference between morning and evening administration. Meanwhile, the development of pulsatile‐release formulations of montelukast represents a pharmaceutical approach to chronotherapy, designed to optimize drug release to provide greater coverage during the early‐morning period when asthma and related allergic symptoms are more prevalent [82]. However, such studies primarily provide evidence for the feasibility of time‐targeted drug delivery and pharmacodynamic coverage and cannot substitute for clinical trials directly comparing administration phases. Thus, current evidence suggests that the leukotriene pathway has a potential basis for chronotherapeutic intervention, but has not yet identified a reproducible and generalizable optimal dosing time. The available evidence is therefore insufficient to support routine adjustment of the timing of montelukast or other leukotriene pathway inhibitors according to circadian phase in current clinical practice.

In addition to ICS, systemic corticosteroids represent one of the anti‐inflammatory treatment classes with relatively substantial evidence supporting a chronopharmacological effect. However, this evidence is derived primarily from early small‐scale studies rather than large contemporary clinical trials in asthma. An early randomized crossover study in patients with nocturnal asthma found that a single dose of prednisone administered at 3:00 p.m. more effectively attenuated the nocturnal decline in FEV_1_ than administration at 8:00 a.m. or 8:00 p.m., while also reducing nocturnal blood eosinophil counts and inflammatory cell infiltration in bronchoalveolar lavage fluid. These findings suggested that afternoon administration may more effectively cover the subsequent nocturnal inflammatory peak [83]. This observation is broadly consistent with the emerging pattern from ICS chronotherapy studies, in which the afternoon‐to‐evening period may represent a biologically relevant therapeutic window. However, systemic corticosteroids are currently used primarily as short courses for acute exacerbations or, in selected patients with severe disease, as maintenance therapy [84], and existing studies of dosing time have not established an evidence base sufficient to modify current treatment strategies. Moreover, cumulative exposure to systemic corticosteroids is associated with multiple short‐ and long‐term adverse outcomes, making minimization of unnecessary exposure a central consideration in clinical use rather than optimization of dosing time alone [84]. Accordingly, the principal value of these studies at present is to demonstrate that the therapeutic effects of systemic corticosteroids may also be temporally dependent and to provide insight into the temporal window of glucocorticoid responsiveness, rather than to establish a new routine dosing regimen.

Unlike small‐molecule therapies, biologics targeting IgE, IL‐5/IL‐5R, IL‐4R*α*, and other inflammatory pathways generally have prolonged durations of action. Accordingly, existing clinical studies have focused primarily on dosing intervals, disease phenotypes, and long‐term disease control, whereas randomized clinical trials directly comparing administration at different circadian phases are currently scarce. The chronopharmacological rationale for these agents is derived mainly from rhythmic features of their molecular targets and effector cells. For example, mast cells and Fc*ε*RI‐mediated IgE responses exhibit marked diurnal variation, suggesting the existence of potential temporal windows for IgE‐mediated allergic responses [85]. In addition, a 2025 study reported altered expression of clock proteins, including CLOCK, BMAL1, REV‐ERB, and ROR, in eosinophils from patients with asthma. Pharmacological modulation of ROR signaling was able to reset the molecular clock in eosinophils while suppressing their migration and exerting anti‐inflammatory and bronchoprotective effects [56]. These findings further support a temporal biological basis for the IL‐5/IL‐5R–eosinophil axis. However, it is important to distinguish between rhythmicity of a therapeutic target and the clinical need to administer a biologic at a specific circadian phase. There is currently insufficient evidence that adjusting the timing of omalizumab, mepolizumab, benralizumab, or other biologics can further improve AHR. Thus, biologics may currently be more appropriately considered as a potential avenue for precision stratification based on circadian phase and inflammatory rhythms rather than as established chronotherapeutic interventions.

Overall, current evidence indicates that, apart from ICS, other established asthma therapies have not yet yielded a mature framework for time‐specific administration. Compared with ICS, therefore, chronotherapy with other treatments is better viewed as a biologically plausible but clinically unvalidated avenue toward precision therapy. Future research should move beyond simple morning‐versus‐evening comparisons toward individualized temporal treatment strategies that integrate sleep phenotype, disease circadian phase, inflammatory phenotype, and pharmacokinetic characteristics of specific therapies. Such approaches should ultimately be evaluated using clinically meaningful outcomes, including nocturnal AHR, 24 h pulmonary function, and acute exacerbations (Table 1).

**TABLE 1:** Human evidence for time‐dependent airway hyperresponsiveness and time‐based treatment strategies in asthma.

Assessment or intervention	Evidence source and population	Timing comparison or protocol	AHR or clinical endpoint and main finding	Evidence directness and certainty^a^	Principal limitation	References
A. Direct human evidence for time‐dependent AHR						
Repeated 24 h histamine or methacholine challenge	Repeated within‐subject bronchial challenge; 9 patients with mild asthma and nocturnal symptoms	Histamine or methacholine challenge every 4 h across 13 consecutive time points	All participants showed reproducible 24 h variation in PC_20_; peak responsiveness occurred at approximately 03:00, whereas a significant FEV_1_ rhythm was present in only 3 participants.	Direct human AHR evidence; low certainty	Very small, selected sample; no circadian‐phase marker; early challenge methods; possible repeated‐test effects	[4]
Night‐versus‐afternoon direct and indirect challenge	Repeated within‐subject challenge; 16 nonsmoking patients with atopic asthma	AMP, methacholine, and propranolol challenge at 16:00 and 04:00	Among patients with diurnal PEF variability ≥ 15%, PC_20_ at 04:00 was lower by 2.3 doubling concentrations for AMP and 0.9 for methacholine; AMP variation correlated with PEF variability (*r* = 0.81).	Direct human AHR evidence; low certainty	Small sample and subgroup analysis; clock time rather than biological phase; airway mediators were not measured	[5]
Repeated histamine and cold‐air hyperventilation challenge	Repeated within‐subject challenge; 15 children with asthma	Testing every 6 h with histamine and hyperventilation using cold, dry air	Histamine responsiveness was greatest at 04:00, whereas cold‐dry‐air responsiveness was greatest at 16:00, demonstrating stimulus‐specific temporal patterns.	Direct human AHR evidence; low certainty	Small pediatric cohort; no circadian‐phase marker; limited control of behavioral and environmental factors	[26]
Night‐versus‐afternoon histamine challenge in children	Repeated within‐subject challenge; 18 children with asthma, stratified by nocturnal airflow limitation	Histamine challenge at 16:00 and 04:00	Histamine PC_20_ was lower at 04:00 in both groups despite markedly different nocturnal changes in FEV_1_, indicating that AHR variation was not simply an extension of baseline airway narrowing.	Direct human AHR evidence; low certainty	Small pediatric groups; only two clock times; no biological‐phase marker or modern confounder control	[27]
Morning‐versus‐evening allergen challenge	Within‐subject allergen challenge; 10 patients with asthma	Identical allergen challenge in the morning and evening; methacholine responsiveness assessed 24 h later	Evening challenge produced more frequent, earlier, and larger late asthmatic responses and greater subsequent methacholine responsiveness.	Direct human challenge evidence; low certainty	Very small sample; complex carryover between allergen and methacholine responses; clock time only	[28]
Daytime repeated methacholine challenge	Repeated methacholine challenge; 24 patients with asthma	PD_20_ compared at 08:00, 14:00, and 20:00; repeated tests separated by ≤ 24 h were also examined	No significant PD_20_ difference was detected within routine daytime hours; short‐interval repetition increased PD_20_, consistent with refractoriness.	Direct negative human AHR evidence; low certainty	Nighttime and early‐morning windows were not studied; repeated‐test refractoriness could mask temporal differences	[17]
B. Supportive human circadian physiology and contemporary diagnostic evidence						
Controlled circadian physiology without bronchial challenge	Controlled human physiology using constant‐routine and forced‐desynchrony protocols in asthma	Behavioral and environmental cycles were controlled or distributed across circadian phases	FEV_1_ reached its nadir and airway resistance and rescue‐inhaler use peaked during the biological night, supporting endogenous circadian regulation of asthma‐related airway status.	Supportive human circadian evidence; moderate certainty for airway function, indirect for AHR	Bronchial challenge was not repeated across circadian phases; therefore, endogenous circadian AHR was not established	[7]
Routine morning‐versus‐afternoon challenge testing	RADicA clinical cohort; different participants tested at different routine clinical times	Morning versus afternoon methacholine and mannitol testing	No significant time‐of‐day difference in challenge positivity was observed (methacholine OR 0.88; mannitol OR 1.40 for afternoon vs. morning).	Indirect contemporary human AHR evidence; low certainty	Between‐subject comparison; routine working hours only; no nighttime/early‐morning testing or circadian‐phase assessment	[8]
C. Human evidence for chronotherapy and treatment timing						
Once‐daily afternoon inhaled corticosteroid dosing	Randomized clinical trials of inhaled corticosteroid timing in asthma	Afternoon dosing (15:00 or 17:30) compared with morning dosing or four‐times‐daily treatment	Afternoon once‐daily administration produced efficacy comparable to four‐times‐daily treatment and generally greater improvement than morning dosing in PEF and related clinical measures.	Human randomized evidence; moderate certainty for clinical timing, low for AHR	Small, short, older trials; formulation‐specific; bronchial challenge was not the principal endpoint	[70, 71]
Morning‐versus‐evening inhaled corticosteroid dosing	Randomized trials of ciclesonide, fluticasone furoate, and fluticasone furoate/vilanterol	Once‐daily morning versus evening administration	Most overall efficacy outcomes were comparable; isolated advantages, such as greater morning PEF after evening ciclesonide, were not consistent across formulations.	Human randomized evidence; moderate certainty against a universal clock‐time rule	Heterogeneous formulations and endpoints; limited circadian phenotyping; no direct AHR outcomes	[72, 73, 74, 75, 76]
Afternoon beclometasone dosing	Randomized three‐period crossover trial; mild‐to‐moderate atopic asthma	Three beclometasone‐dipropionate dosing schedules, including once‐daily afternoon administration	All schedules improved asthma control and FeNO; afternoon dosing most clearly attenuated the 22:00 FEV_1_ decline and nocturnal/early‐morning blood eosinophil rise, without altering serum cortisol.	Relatively strong human randomized evidence; moderate certainty for nocturnal clinical outcomes, low for AHR	Short duration, selected phenotype, single ICS; no repeated bronchial challenge endpoint	[10]
Evening‐versus‐morning inhaled corticosteroid dosing	Systematic review and meta‐analysis of once‐daily ICS timing studies	Evening versus morning administration	Evening dosing produced a modest improvement in lung function, but effects varied across studies.	Secondary human evidence; low‐to‐moderate certainty	Clinical and methodological heterogeneity; mainly older trials; direct AHR outcomes were limited	[78]
Twenty‐four‐hour LABA/LAMA regimens	Randomized pharmacodynamic trials in symptomatic asthma	Once‐daily or twice‐daily regimens designed to provide 24 h bronchodilator coverage	Sustained bronchodilation was demonstrated, but a reproducible circadian‐phase advantage was not established.	Human randomized but indirect timing evidence; low certainty for chronotherapy	Primarily compared dose frequency and coverage rather than biological phase; no direct AHR endpoint	[79, 80]
Morning‐versus‐evening montelukast dosing	Randomized crossover study in children with exercise‐induced bronchoconstriction	Morning versus evening administration	Both schedules reduced the post‐exercise fall in FEV_1_, with no significant difference between dosing times.	Human randomized challenge evidence; low‐to‐moderate certainty for this phenotype	Pediatric, phenotype‐specific study; does not establish a general optimal phase for asthma or AHR	[81]
Afternoon prednisone dosing	Randomized crossover study in nocturnal asthma	Prednisone at 15:00 compared with 08:00 or 20:00	15:00 administration more effectively attenuated the nocturnal decline in FEV_1_ and reduced nocturnal blood and bronchoalveolar inflammatory‐cell responses.	Human randomized evidence; low‐to‐moderate certainty for nocturnal outcomes, indirect for AHR	Small, early study; systemic corticosteroid risks; no direct bronchial‐challenge endpoint	[83]

*Note:* Evidence interpretation. Direct human AHR evidence required standardized bronchial challenge testing across more than one time point within the same participants. Supportive human evidence included controlled circadian physiology or time‐of‐day diagnostic studies without repeated within‐subject bronchial challenge. Treatment‐timing evidence was judged separately for clinical outcomes and for direct AHR outcomes.

Abbreviations: AHR, airway hyperresponsiveness; AMP, adenosine 5′‐monophosphate; FEV_1_, forced expiratory volume in 1 s; ICS, inhaled corticosteroid; LABA, long‐acting *β*_2_‐agonist; LAMA, long‐acting muscarinic antagonist; OR, odds ratio; PC_20_, provocative concentration causing a 20% fall in FEV_1_; PD_20_, provocative delivered dose causing a 20% fall in FEV_1_; PEF, peak expiratory flow.

^a^ Certainty. Certainty is a qualitative narrative judgment used in this review rather than a formal GRADE rating. It considers design, directness to human time‐dependent AHR, sample size, circadian control, consistency, and clinical applicability.

### 5.3 Clock‐Targeted and Circadian Alignment Strategies

Beyond optimizing the timing of existing therapies, further development of asthma chronotherapy may involve two broad strategies. One is to directly target clock‐related molecules to reshape dysregulated local circadian networks, whereas the other is to reduce phase misalignment between central and peripheral clocks through the management of sleep, light exposure, and behavioral rhythms. Importantly, neither strategy has yet generated randomized clinical evidence specifically targeting AHR. Their current significance therefore lies primarily in providing directions for future precision chronotherapy rather than in supporting established therapeutic recommendations.

Direct targeting of circadian pathways offers a potential means of intervening in asthma at an upstream level. Increasing evidence indicates that alterations in molecular clock components, including REV‐ERB, ROR, and BMAL1, are involved not only in immune‐cell function, airway epithelial homeostasis, and glucocorticoid responsiveness, but also in disease severity and allergic status, highlighting their potential druggability [36, 56, 86]. Accordingly, a growing number of pharmacological tool compounds targeting clock‐related pathways have been developed. For example, in an in vitro study using human bronchial epithelial cells, Duraisamy et al. [87] found that the REV‐ERBα agonist SR10067 attenuated Th2 cytokine‐induced epithelial barrier dysfunction, as reflected by reduced barrier permeability, improvement of tight‐junction abnormalities, and attenuation of inflammatory readouts. This study provided direct evidence that REV‐ERB*α* activation can modulate alterations in epithelial barrier function. Because epithelial barrier disruption not only facilitates the entry of allergens and pollutants into downstream immune networks but may also amplify the development of nocturnal airway hyperresponsiveness [88], these findings provide relatively direct support for therapeutically targeting the molecular clock–epithelial barrier interface.

In addition to SR10067, another class of clock‐targeting research compounds warrants attention. The ROR ligand SR1001 has been shown to reset the molecular clock in eosinophils in vitro, suppress eosinophil migration, and exert anti‐inflammatory and bronchoprotective effects in animal models of allergic airway inflammation [56]. Collectively, these findings suggest that pharmacological modulation of dysregulated molecular clocks may represent an important future therapeutic direction. However, the available pharmacological evidence remains derived predominantly from cellular and animal studies, while the specificity, potential off‐target effects, long‐term safety, and human pharmacokinetic properties of these compounds have not been sufficiently established. Research tool compounds should therefore not be equated with established clinical drug candidates. At present, direct clock‐targeted therapy is more appropriately regarded as a strategy for mechanistic validation and drug development rather than as an established clinical treatment.

Compared with direct pharmacological targeting of the molecular clock, another potential approach—circadian alignment—may be more readily translated into clinical practice, particularly through the management of sleep and light exposure. At the level of sleep, a nationwide cross‐sectional study of US adults based on the National Health and Nutrition Examination Survey (NHANES) published in 2019 found that abnormal sleep duration was significantly associated with lower lung function. Short sleep duration was also associated with a higher prevalence of asthma and greater bronchodilator responsiveness. Although the study had several limitations, its findings highlighted a potential link between sleep and asthma and provided a basis for subsequent prospective investigations [89]. More recently, a prospective cohort study demonstrated associations between sleep duration and clinical and inflammatory features of asthma, suggesting that sleep duration may represent a potentially modifiable factor of clinical relevance to asthma management [90]. Similarly, a meta‐analysis incorporating 23 studies reported a positive association between sleep disturbances and asthma, although the quality of evidence was considered low because of potential sources of bias [91]. Collectively, these findings provide biological rationale for incorporating sleep management into asthma care. However, because the available evidence is derived predominantly from observational studies, subjective bias cannot be completely excluded, and interventional studies remain limited, sleep management cannot yet be regarded as an in vivo–validated therapeutic approach for AHR. In addition, light exposure, as the most important environmental time cue, is a key regulator of sleep and behavioral rhythms [92]. Nevertheless, from an evidence‐level perspective, randomized trials centered on light‐based interventions and directly evaluating asthma AHR or 24 h lung function are still lacking. Both preclinical and clinical investigations are therefore needed to clarify the therapeutic potential of light‐based circadian interventions. Overall, sleep and light management have a plausible basis for clinical translation, but they cannot yet be considered standardized therapeutic strategies applicable to all patients with asthma.

Individual variability is another important consideration, as individuals may respond differently to the same circadian misalignment challenge. Chronotype and sex are among the potentially important modifiers of these responses. Chronotype is a behavioral phenotype reflecting an individual's preferred timing of sleep and wakefulness, and its association with asthma risk has been demonstrated in a large population‐based study. Using data from more than 280,000 UK Biobank participants, Maidstone et al. [93] found that individuals with a morning chronotype who engaged in irregular shift work involving night shifts had a 55% higher risk of moderate‐to‐severe asthma (OR 1.55, 95% CI 1.06–2.27). This finding may reflect the fact that individuals with a morning chronotype have an earlier endogenous circadian phase and therefore experience greater circadian misalignment during night‐shift work [94]. By contrast, the circadian phase of evening‐type individuals may be relatively more compatible with night‐shift schedules, potentially conferring greater tolerance to night work than in morning‐type individuals [95]. These observations suggest that chronotype may modify inflammatory and physiological responses to circadian disruption. It is therefore plausible that chronotype could also modify the response to circadian alignment interventions, although this possibility remains to be tested in interventional studies. With respect to sex, a 2025 UK Biobank study reported that women engaged in long‐term night‐shift work had higher odds of moderate‐to‐severe asthma (OR 1.50, 95% CI 1.18–1.91), whereas a similar association was not observed in men; this pattern was particularly evident among postmenopausal women who had not received hormone therapy [96]. The physiological basis of this sex difference may involve interactions between estrogen and the pulmonary circadian system [97]. Taken together, these observations indicate that factors such as chronotype, sex, and hormonal status may modify the physiological consequences of circadian misalignment and, consequently, the response to circadian alignment strategies. Future clinical circadian interventions should therefore consider individual endocrine characteristics and chronotype when developing personalized approaches to circadian realignment.

Taken together, the available evidence provides a rationale for moving asthma chronotherapy beyond fixed clock‐time schedules toward a more biologically phase‐oriented approach. Strategies that align medication administration and circadian interventions with an individual's endogenous biological phase may be particularly relevant to patients with nocturnal asthma, pronounced diurnal variation in lung function, social jetlag, or shift‐work exposure. However, it is important to emphasize that current evidence remains largely at the level of association and mechanistic exploration. Direct human evidence demonstrating that circadian interventions tailored according to chronotype, biological phase, or sex can improve AHR or lung function or reduce acute exacerbations remains lacking. Thus, direct targeting of the molecular clock and personalized circadian alignment represent promising directions for future research into precision chronotherapy for asthma, but neither approach currently provides a sufficiently established evidence base for incorporation into standardized clinical treatment guidelines (Table 2).

**TABLE 2:** Preclinical and translational clock‐related strategies relevant to airway hyperresponsivenes**s**.

Target or strategy	Evidence source and model	Clock or temporal manipulation	Main finding relevant to AHR	Evidence category and certainty^a^	Principal translational limitation	References
REV‐ERBα–muscarinic receptor axis	HDM allergic‐airway mouse model and precision‐cut lung slices	Time‐of‐day assessment and loss of REV‐ERB*α*	AHR and ex vivo methacholine contraction were time dependent; REV‐ERB*α* loss abolished rhythmic AHR and rhythmic Chrm1/Chrm3 expression.	Preclinical mechanistic evidence; moderate within model, very low for human translation	No direct validation of the REV‐ERB*α*–cholinergic–AHR causal chain in human asthma	[33]
BMAL1–DUSP4–p38 MAPK–GR axis	HDM asthma model with airway epithelial BMAL1 disruption	Genetic disruption of BMAL1 and pharmacological DUSP4 inhibition	BMAL1 deficiency worsened inflammation and glucocorticoid resistance; DUSP4–p38 MAPK signaling linked BMAL1 to GR phosphorylation, and DUSP4 inhibition restored steroid sensitivity.	Preclinical mechanistic evidence; moderate within model, very low for clinical use	Does not demonstrate phase‐specific AHR improvement in humans; target specificity and safety are unknown	[41]
Club‐cell BMAL1 and epithelial–immune timing	Chronic HDM allergic‐airway mouse model	Selective deletion of Bmal1 in airway club cells	Deletion abolished diurnal rhythms of airway permeability, pulmonary eosinophil accumulation, and Th2‐cell recruitment.	Preclinical mechanistic evidence; moderate for temporal epithelial–immune coupling, indirect for AHR	Primary endpoints were barrier function and cell recruitment rather than AHR; no human intervention data	[50]
Melatonin signaling	Human ASM in vitro; ozone‐aggravated and allergic‐asthma mouse models; partial validation in primary human ILC2s	MT_2_‐receptor activation or melatonin treatment	Pharmacological melatonin enhanced ASM contraction in vitro, whereas animal studies showed TRPV1–NRF2‐ and ILC2–glutathione‐dependent protection against inflammation and AHR.	Translational and preclinical evidence; low certainty because effects are context dependent	Bidirectional effects, pharmacological concentrations, and absent phase‐specific human AHR trials preclude therapeutic recommendations	[45, 46, 47]
ROR modulation in eosinophils	Peripheral‐blood eosinophils from patients with asthma plus preclinical allergic‐airway models	Pharmacological resetting of the eosinophil clock with an ROR ligand	ROR modulation reset eosinophil clock features, suppressed migration, and produced anti‐inflammatory and bronchoprotective effects in preclinical models.	Human‐cell and preclinical evidence; low certainty for circadian AHR	Peripheral rather than airway eosinophils; research compound; no phase‐specific dosing trial or direct human AHR outcome	[56]
PER1/BMAL1–SOCE/Ca^2+^ coupling in ASM	Developing human airway smooth‐muscle cells under hyperoxia	Manipulation or restoration of PER1 and BMAL1	Clock‐protein manipulation altered store‐operated Ca^2+^ entry and histamine‐induced intracellular Ca^2+^ responses, supporting coupling between clock status and contractile signaling.	Human‐cell mechanistic support; very low certainty for adult asthma AHR	Developmental hyperoxia model rather than adult asthma; no 24 h AHR phenotype or clinical intervention	[57]
BMAL1–redox–ferroptosis pathway	HDM asthma model and human bronchial epithelial cells	BMAL1 deficiency and pharmacological ferroptosis inhibition	BMAL1 loss increased ROS, lipid peroxidation, and airway injury with reduced GPX4/SLC7A11; ferroptosis inhibition partially reversed inflammation and remodeling.	Preclinical mechanistic evidence; moderate for oxidative injury, very low for temporal human AHR	Circadian‐phase dependence of redox injury and AHR was not directly tested in humans	[69]
REV‐ERBα agonist SR10067	Human bronchial epithelial cells in vitro	Pharmacological REV‐ERB*α* activation	SR10067 attenuated Th2‐cytokine‐induced barrier permeability, tight‐junction abnormalities, and inflammatory readouts.	Human‐cell mechanistic evidence; very low certainty for clinical efficacy	No animal or human asthma trial; barrier endpoints rather than AHR; off‐target effects and pharmacokinetics remain undefined	[87]
Pulsatile‐release montelukast formulation	Formulation development with in vitro and in vivo pharmacokinetic evaluation	Delayed release designed to cover the early‐morning vulnerability window	Demonstrated feasibility of time‐targeted drug delivery and temporal drug exposure.	Preclinical formulation evidence; very low certainty for clinical benefit	No clinical phase‐comparison trial and no evidence that the formulation improves AHR or asthma outcomes	[82]
Circadian‐alignment strategies	Human observational cohorts, meta‐analysis, and non‐asthma circadian intervention evidence	Sleep regularity, light exposure, shift‐work reduction, and chronotype‐aware alignment	Sleep and circadian misalignment are associated with asthma risk, lung function, and inflammatory features; chronotype and sex may modify susceptibility.	Indirect human evidence and hypothesis‐generating strategy; very low certainty for AHR treatment	Predominantly observational; confounding and reverse causality; no randomized asthma trial with direct AHR outcomes	[89, 90, 91, 92, 93, 94, 95, 96]

*Note:* Evidence interpretation. Preclinical mechanistic evidence includes animal, ex vivo, and in vitro studies that manipulate clock components or temporal signals. Human‐cell findings support biological plausibility but do not establish efficacy or circadian mediation of AHR in patients.

Abbreviations: AHR, airway hyperresponsiveness; ASM, airway smooth muscle; BMAL1, brain and muscle ARNT‐like protein 1; Chrm1/Chrm3, cholinergic receptor muscarinic 1 and 3, respectively; DUSP4, dual‐specificity phosphatase 4; GPX4, glutathione peroxidase 4; GR, glucocorticoid receptor; HDM, house dust mite; ILC2, group 2 innate lymphoid cell; MAPK, mitogen‐activated protein kinase; NRF2, nuclear factor erythroid 2‐related factor 2; ROR, retinoic acid receptor‐related orphan receptor; SLC7A11, solute carrier family 7 member 11; SOCE, store‐operated calcium entry; TRPV1, transient receptor potential vanilloid 1.

^a^ Certainty. The certainty descriptor refers to the specific conclusion shown in the row. A finding can be well supported within an experimental model while remaining very uncertain for human clinical translation.

## 6 Limitations and Future Directions

The current evidence on the temporal dependence of AHR remains constrained by the limited number of studies, heterogeneity in experimental designs, and variability in outcome assessment. Direct comparisons of bronchial challenge responses at different time points in humans remain relatively scarce and have generally been based on small, early studies, with some investigations covering only limited daytime or nighttime periods. Most studies have not simultaneously assessed circadian phase markers, such as melatonin or core body temperature, nor have they rigorously controlled for sleep, posture, physical activity, food intake, environmental exposures, and medication timing. Consequently, it remains difficult to distinguish endogenous circadian effects from differences in clock time attributable to behavioral or environmental factors. In addition, substantial heterogeneity exists in the bronchoconstrictor stimuli, delivery devices, inhalation protocols, medication washout periods, and AHR endpoints used across studies. Patient characteristics, including age, asthma phenotype or endotype, disease severity, and treatment status, have also rarely been adequately stratified, limiting the comparability and generalizability of the available findings.

A major limitation of current mechanistic research is that the evidence chain has not yet been established in humans. Existing studies often demonstrate separately that a given signaling pathway contributes to asthma‐related inflammation, ASM contraction, or general circadian regulation, but such evidence alone cannot establish that the same pathway mediates the diurnal variation in human AHR. Similar challenges exist in clinical translation. Although studies of ICS administration timing include some randomized clinical evidence, most have used lung function, symptoms, or inflammatory markers as primary outcomes, while direct assessments of AHR remain limited. Chronotherapy involving other asthma medications, direct targeting of clock pathways, and sleep‐ or light‐based interventions remain largely at the observational or preclinical stage. Therefore, current evidence is insufficient to define a universally applicable optimal time for AHR assessment or treatment and does not yet support considering candidate clock‐related pathways as established therapeutic targets.

Future studies should prioritize human chronobiological designs with clearly defined circadian phases and rigorous control of behavioral factors. Standardized bronchial challenge testing should be performed repeatedly across a complete 24 h cycle or at multiple circadian phases, with complementary AHR endpoints, including the provocative dose causing a 20% fall in FEV_1_ (PD_20_), dose–response slope, and maximum airway narrowing. These assessments should be accompanied by measurements of circadian phase, baseline lung function, airway inflammation, and relevant mechanistic biomarkers. Study populations should be prospectively stratified according to chronotype, sex, age, asthma phenotype or endotype, disease severity, nocturnal symptoms, shift‐work exposure, and treatment status. Mechanistic studies should integrate temporal phenotyping, molecular pathway measurements, and pathway‐specific interventions within the same experimental framework to determine whether candidate pathways truly mediate temporal changes in AHR. Clinical trials should, in turn, incorporate direct AHR outcomes together with clinically meaningful long‐term endpoints to evaluate phase‐oriented dosing strategies. Such studies should first identify the patient characteristics associated with differential responses to treatment timing and then determine whether these findings can be translated into feasible individualized management strategies.

## 7 Conclusion

Current evidence indicates that AHR can manifest as a dynamic physiological phenotype that varies with the timing of assessment. Several repeated bronchial challenge studies in humans have observed enhanced airway responsiveness from nighttime to the early morning, and this variation does not appear to fully parallel the temporal changes in baseline lung function. Neuroendocrine and autonomic regulation, epithelial–immune temporal coupling, ASM reactivity, and metabolic and redox homeostasis may collectively influence baseline airway caliber, inflammatory mediator burden, and contractile response gain, thereby contributing to temporal differences in AHR. Emerging chronotherapy studies further suggest that the timing of medication administration may influence treatment responses in some patients with asthma, with ICS currently supported by relatively more substantial human evidence, whereas chronotherapy involving other medications and clock‐related interventions remains investigational. Overall, the available evidence is not yet sufficient to attribute temporal variation in AHR broadly to endogenous circadian rhythms, nor does it support a universal optimal dosing time for all patients with asthma. Rather, the key implication of the current evidence is that time should be recognized as an important dimension to be systematically recorded and validated in the assessment of AHR, the interpretation of its underlying mechanisms, and the evaluation of treatment responses, thereby providing a foundation for future development of biologically phase‐oriented and individualized asthma management.

## Author Contributions

**Chang Zhou:** writing – original draft, conceptualization. **Xinyue Hu:** writing – original draft. **Lijuan Zeng:** validation, supervision. **Yi Tang:** visualization. **Chuxian Yi:** writing – review and editing. **Guomin Zhang:** writing – review and editing, funding acquisition. **Sujuan Peng:** writing – review and editing, Funding acquisition.

## Funding

This work was supported by the Graduate Research and Innovation Project of Hunan Province (Grant CX20251186; Chang Zhou) and the Undergraduate Research and Innovation Fund of Hunan University of Chinese Medicine (Grants 2025BKS059 and 2025BKS131; Xinyue Hu and Yi Tang).

## Ethics Statement

The authors have nothing to report.

## Consent

The authors have nothing to report.

## Conflicts of Interest

The authors declare no conflicts of interest.

## Supporting information


**Table S1:** PubMed search strategy. The following search strategy was developed using PubMed as an example. Equivalent adaptations were made for Web of Science Core Collection, Embase, and Scopus according to each database’s controlled vocabulary, field tags, and syntax. The final search was combined as #1 AND #3 AND (#2 OR #4 OR #5).

## Data Availability

Data sharing not applicable to this article as no datasets were generated or analyzed during the current study.
