# Supplementary material for: Time‐of‐Day Variation in Airway Hyperresponsiveness in Asthma: Potential Circadian Mechanisms and Translational Implications
Source: Clin Transl Allergy. 2026 Nov 7;16(11):e70221. doi: 10.1002/clt2.70221 (PMC13744419; doi:10.1002/clt2.70221)
Supplement: Supplementary file 1 [file CLT2-16-e70221-s001.docx]

**Supplementary Material, Table S1. PubMed search strategy.**

The following search strategy was developed using PubMed as an example. Equivalent adaptations were made for Web of Science Core Collection, Embase, and Scopus according to each database’s controlled vocabulary, field tags, and syntax. The final search was combined as #1 AND #3 AND (#2 OR #4 OR #5).

| **Search module** | **PubMed search strategy** |
| --- | --- |
| **#1 Asthma** | ("Asthma"[Mesh] OR asthma*[Title/Abstract]) |
| **#2 Airway hyperresponsiveness (AHR) and bronchial challenge** | ("Bronchial Hyperreactivity"[Mesh] OR "Bronchial Provocation Tests"[Mesh] OR "airway hyperresponsiveness"[Title/Abstract] OR "airway responsiveness"[Title/Abstract] OR "bronchial hyperresponsiveness"[Title/Abstract] OR "bronchial challenge"[Title/Abstract] OR "bronchial provocation"[Title/Abstract] OR methacholine[Title/Abstract] OR histamine[Title/Abstract] OR mannitol[Title/Abstract] OR "adenosine monophosphate"[Title/Abstract] OR AMP[Title/Abstract] OR "hypertonic saline"[Title/Abstract] OR "cold air"[Title/Abstract] OR exercise[Title/Abstract] OR "eucapnic voluntary hyperpnea"[Title/Abstract] OR "eucapnic voluntary hyperventilation"[Title/Abstract] OR "allergen challenge"[Title/Abstract] OR PC20[Title/Abstract] OR PD20[Title/Abstract]) |
| **#3 Circadian rhythm and time** | ("Circadian Rhythm"[Mesh] OR circadian[Title/Abstract] OR diurnal[Title/Abstract] OR "time of day"[Title/Abstract] OR time-dependent[Title/Abstract] OR "biological clock"[Title/Abstract] OR "biological night"[Title/Abstract] OR "circadian phase"[Title/Abstract] OR chronotype[Title/Abstract] OR melatonin[Title/Abstract] OR "dim-light melatonin onset"[Title/Abstract] OR "constant routine"[Title/Abstract] OR "forced desynchrony"[Title/Abstract] OR nocturnal[Title/Abstract]) |
| **#4 Biological mechanisms** | ("clock gene"[Title/Abstract] OR "clock genes"[Title/Abstract] OR BMAL1[Title/Abstract] OR ARNTL[Title/Abstract] OR CLOCK[Title/Abstract] OR PER1[Title/Abstract] OR PER2[Title/Abstract] OR CRY1[Title/Abstract] OR CRY2[Title/Abstract] OR REV-ERB[Title/Abstract] OR NR1D1[Title/Abstract] OR ROR[Title/Abstract] OR glucocorticoid*[Title/Abstract] OR cortisol[Title/Abstract] OR cholinergic[Title/Abstract] OR adrenergic[Title/Abstract] OR eosinophil*[Title/Abstract] OR "mast cell"[Title/Abstract] OR "mast cells"[Title/Abstract] OR cytokine*[Title/Abstract] OR "airway smooth muscle"[Title/Abstract] OR epitheli*[Title/Abstract]) |
| **#5 Chronotherapy** | ("Chronotherapy"[Mesh] OR chronotherap*[Title/Abstract] OR chronopharmacolog*[Title/Abstract] OR "dosing time"[Title/Abstract] OR "administration time"[Title/Abstract] OR morning[Title/Abstract] OR evening[Title/Abstract] OR "inhaled corticosteroid"[Title/Abstract] OR "inhaled corticosteroids"[Title/Abstract] OR bronchodilator*[Title/Abstract] OR "leukotriene receptor antagonist"[Title/Abstract] OR "leukotriene receptor antagonists"[Title/Abstract] OR biologic*[Title/Abstract]) |
